# Isoselenocyanates with Sterically Encumbered Substituents—Synthesis, Structures, and Spectroscopic Properties

**DOI:** 10.1021/acs.inorgchem.6c00462

**Published:** 2026-03-16

**Authors:** Vânia D. Schwade, Michael L. Neville, Guilhem Claude, Maximilian Roca Jungfer, Adelheid Hagenbach, Ernesto Schulz Lang, Joshua S. Figueroa, Ulrich Abram

**Affiliations:** † Department of Chemistry, Natural and Exact Sciences, 28118 Federal University of Santa Maria, Av. Roraima, n.1000, Santa Maria 97105-900, RS, Brazil; ‡ Department of Chemistry and Biochemistry, 8784 University of California San Diego, 9500 Gilman Drive, MC 0358, La Jolla, California 92093, United States; § Institute of Chemistry and Biochemistry, Freie Universität Berlin, Fabeckstr. 34/36, D-14195 Berlin, Germany; ∥ Institute for Nuclear Waste Disposal (INE), Karlsruhe Institute of Technology, Hermann-von-Helmholtz-Platz 1, D-76344 Eggenstein-Leopoldshafen, Germany

## Abstract

A series of isoselenocyanates, SeCNR, spanning over a variety of organic residues in terms of steric encumbrance and electronic effects, was synthesized by reactions of corresponding isocyanides with elemental selenium. The residues range from tertiary butyl (^*t*^Bu), phenyl (Ph), 2,4,6-trimethylphenyl (Mesityl), 2,6-diisopropylphenyl (^*i*‑Prop2^Ph), 2,6-di­(2,4,6-trimethylphenyl)­phenyl (Ar^Mes2^), 2,6-di­(2,6-diisopropylphenyl)­phenyl (Ar^Dipp2^), 2,6-di­(2,4,6-triisopropylphenyl)­phenyl (Ar^Tripp2^), and 4-fluorophenyl (PhF) to 2,6-di­{3,5-di­(trifluoromethyl)­phenyl}­4-fluorophenyl (*p*-FAr^DarF2^). They were formed as crystalline solids or viscous oils in medium to good yields, which recommends this synthetic approach as generally suitable. The products were studied by X-ray diffraction and spectroscopic methods, including ^77^Se NMR spectroscopy. The influence of the organic substituents on the ^77^Se NMR chemical shifts of the isoselenocyanates is in good accordance with DFT-modeled values. Experimental ^77^Se–^13^C couplings of 280 Hz could be derived for Se^13^CNAr^Dipp2^ prepared from a sample of ^13^CNAr^Dipp2^ (isotopic enrichment: 99%). A small amount of a selenourea-type product, (*i*-Prop)_2_NC­(Se)­NH*p*-FAr^DarF2^, was isolated from a reaction mixture of CN*p*-FAr^DarF2^ with selenium and diisopropylamine as the supporting base, which indicates an influence of the supporting base used for the course of the reaction.

## Graphical abstract

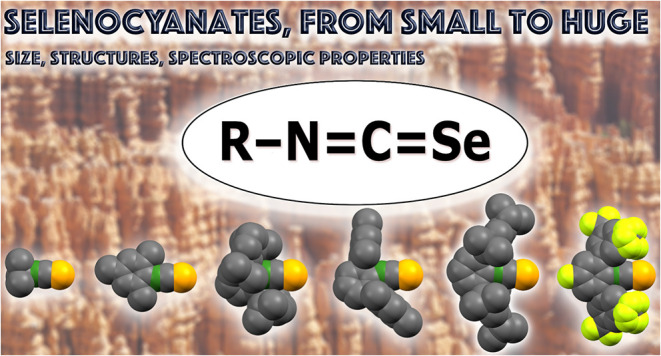

## Introduction

The first report on the discovery of the element selenium in 1818 already contains information about an unexpected “biological/medicinal” effect caused by one of its compounds. Johan Berzelius describes a “stabbing pain in the nose and long-lasting inflammation after inhaling a gas bubble of its hydride, no larger than a pea.”^1^ A more detailed report about the discovery and the early days of selenium can be found in an excellent essay by Trofast.^2^ Now, more than 200 years later, there is still ongoing interest in the chemistry of selenium-containing molecules in different fields of research.^3–8^ A main focus is set to the biological chemistry of this element stimulated by its vital role in glutathione peroxidase, the first identified mammalian selenoprotein^9–12^ and the role of selenium compounds in pharmacology.^13–15^ A potential use in medicinal applications is also discussed for the hitherto relatively little explored class of organoisoselenocyanates.^16–18^

Synthetic access to isoselenocyanates is frequently done by reactions of the corresponding isocyanides with elemental selenium^19–25^ or by reactions of organic halides with potassium selenocyanate.^26–28^ Synthetic approaches starting from amines, organic cyanates or via isomerization of organoselenocyanates are also known.^20,29–33^ However, systematic studies comparing the structures and spectroscopic properties of isoselenocyanates have not yet been reported. This fact stimulated us to synthesize such compounds with organic residues with a large variety of steric bulk and electronic properties. They are depicted in Chart 1 together with their abbreviations used throughout the present paper.

**Chart 1:**
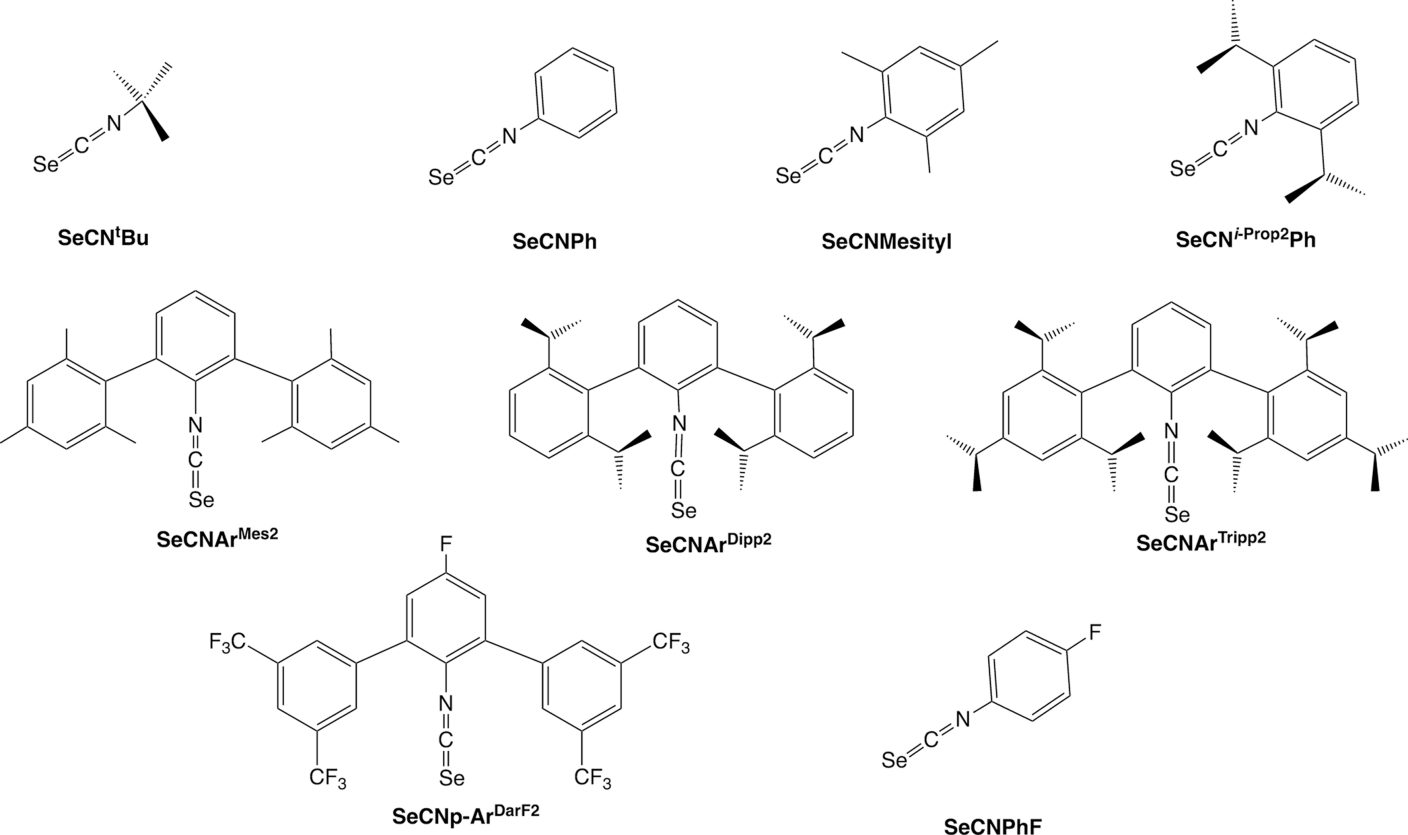
Isoselenocyanates Studied in the Present Paper

## Results and Discussion

The isoselenocyanates shown in Chart 1 were synthesized by reactions of elemental selenium with a moderate excess of the corresponding isocyanides in the presence of NEt_3_ in dry solvents under an atmosphere of dry argon (Scheme 1). The use of an excess amount of the isocyanides for such reactions is somewhat counterintuitive and caused indeed in some cases minor problems during the purification of the products. However, it avoids the formation of selenium-containing side products and allows the measurements of ^77^Se NMR spectra of reasonable quality also in cases of instable isoselenocyanates (e.g., SeCNPh or SeCNPhF), where a gradual decomposition of the isolated products is observed starting shortly after their isolation. Presumably, the use of elemental selenium as the limiting reagent avoids significant production of reactive poly selenium rings and chains, which are known to form upon the dissolution of gray selenium in nucleophilic solvents, and which may react further with incipiently formed isoselenocyanates.^34^ Using this approach, reactions with small isocyanides could be conducted in THF and with relatively short reaction times, while reactions with the sterically more encumbered *m*-terphenylisocyanides required more drastic reaction conditions such as a prolonged reflux in boiling toluene. The individual reaction conditions applied are summarized in Scheme 1.

**Scheme 1:**
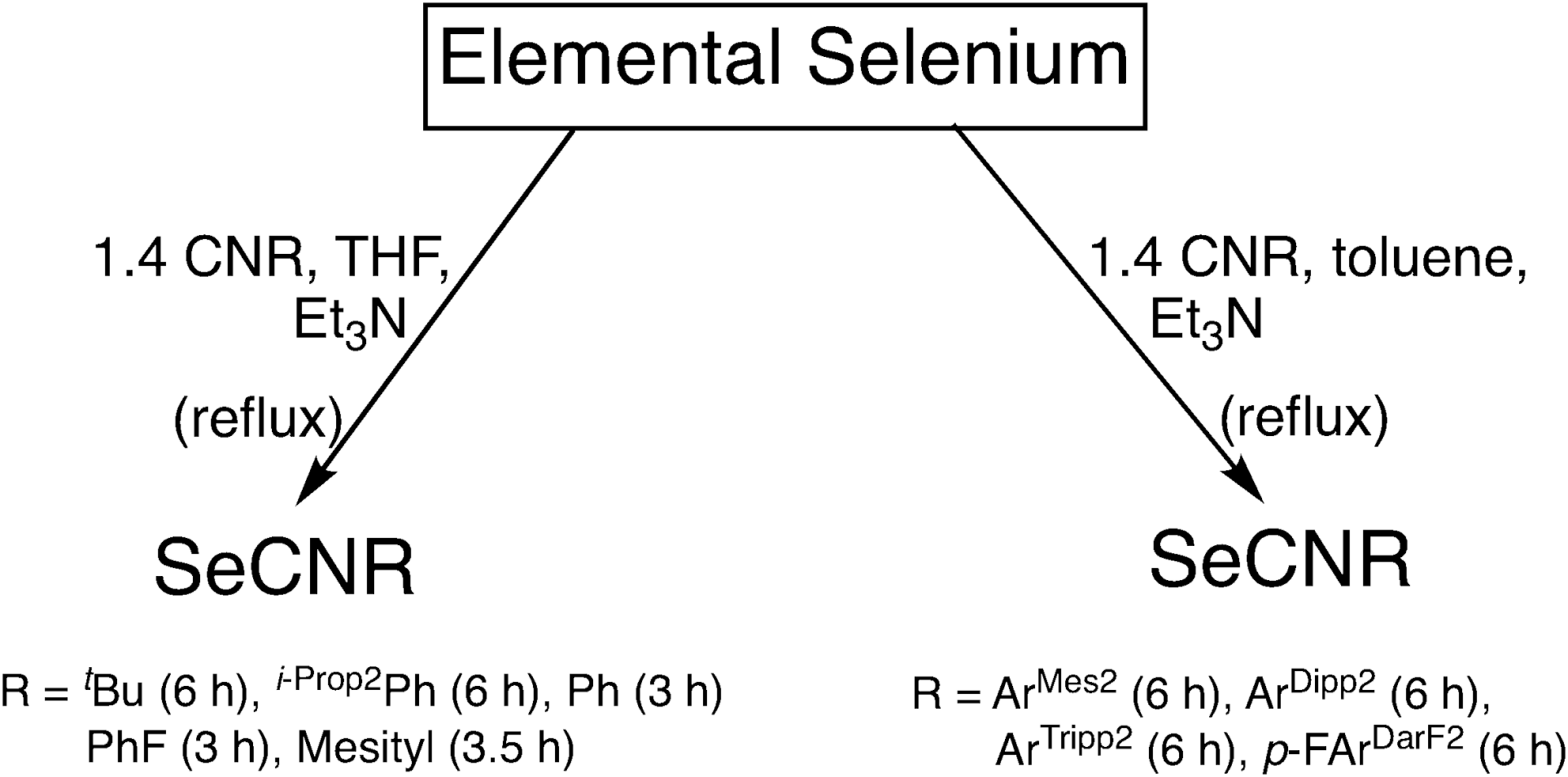
Conditions Applied for the Synthesis of the Novel Isoselenocyanates

Most of the products are colorless solids, which can be obtained directly from the reaction mixtures and purified by recrystallization. SeCN^*i*‑Prop2^Ph, SeCNPh and SeCNPhF, however, are viscous oils, and the latter two compounds decompose already at ambient temperature, which is evident by the gradual appearance of a reddish color and the final formation of elemental selenium.

The isoselenocyanates show intense IR stretches between 2040 and 2130 cm^–1^, which are in the same region as in the parent isocyanides. Their ^1^H NMR spectra are unexceptional and confirm the composition of the organic residues. Interestingly, the ^77^Se NMR chemical shifts in CDCl_3_ span over the relatively large spectral range from −274.1 ppm for SeCN*p*-FAr^DarF2^ to −345.5 ppm for SeCN^*t*^Bu. Figure 1 depicts the recorded spectra. Obviously, the steric bulk and electronic effects of the organic residues seem to influence the ^77^Se chemical shifts (δ). Thus, the lowest shielding of the ^77^Se nuclei with δ values > −300 ppm is observed for the bulky *m*-terphenyl isoselenocyanates SeCNAr^Mes2^, SeCNAr^Dipp2^, and SeCNAr^Tripp2^, but also for such with fluorosubstituted phenyl residues such as SeCN*p*-FAr^DarF2^ and SeCNPhF. Markedly more negative chemical shifts are found for phenylisoselenocyanates with aliphatic substituents such as SeCNMesityl or SeCN^*i*‑Prop2^Ph and particularly for the aliphatic SeCN^*t*^Bu.

**Figure 1:**
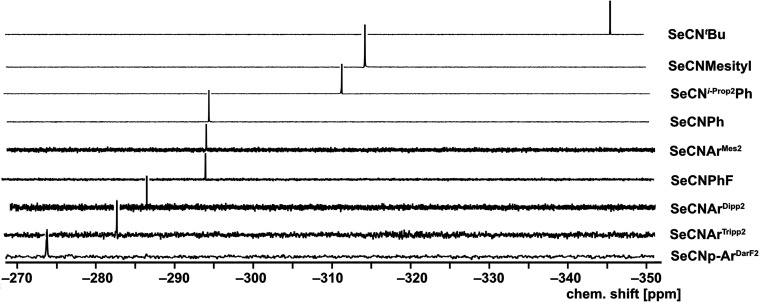
^77^Se NMR spectra of the isoselenocyanates of the present study in CDCl_3_.

The latter trend, the observed high-field shift of the ^77^Se NMR signals due to aliphatic substituents in isoselenocyanates, is confirmed by a few spectra recorded for amino acid derivatives (−356 to – 358 ppm),^22^ but has interestingly also been observed for 1,1-di­(isoselenocyanato)­ferrocene, for which a chemical shift of −365 ppm has been reported.^23^ The chemical shifts depicted in Figure 1 span a range of approximately 70 ppm. This is not particularly large, having in mind the almost 3000 ppm scale of ^77^Se chemical shifts, but nevertheless notable with respect to the fact that the selenium atoms are separated from the organic residues by three bonds. Even between the values observed for the different arylisoselenocyanates, there are chemical shift variations of about 40 ppm, which is the range that has been observed previously for a number of diaryldiselenides, where the selenium atoms are directly bonded to the organic residues.^35^

Couplings between the ^77^Se and the ^13^C nuclei of the isoselenocyanate unit could not be resolved unambiguously in spectra with ^13^C in natural abundance. Hence, we prepared for one representative, SeCNAr^Dipp2^, a sample starting from ^13^CNAr^Dipp2^ (isotopic enrichment: 99%), which allowed an unambiguous assignment of the isoselenocyanate carbon atom and the recording of experimental ^77^Se–^13^C couplings in both the ^77^Se and ^13^C NMR spectra.

The corresponding spectra are depicted in Figure 2 indicating a spin–spin coupling constant between the two nuclei of 280 Hz. This coupling is thus the largest value reported for organoselenium compounds. They accompany the values found for COSe (286.9 Hz) and F_2_CSe (263.3 Hz)^36^ but are larger than in the organoselenocyanates CH_3_SeCN (237.8 Hz) or PhSeCN (239.3 Hz).^37^ Markedly smaller ^77^Se–^13^C couplings are found for selenoacetylenes (≈190 Hz),^37^ Se–C_sp2_ compounds (90–155 Hz)^38–41^ and aliphatic organoselenium compounds (40–80 Hz).^38,42–47^ The obvious dependence of the observed ^77^Se–^13^C couplings on the “s character” of the contributing carbon atoms supports the previously published assumption^36,37^ that such interactions are significantly influenced by the Fermi contact term,^48^ and it would be interesting to have a tighter view to this point with the tools of the modern computational chemistry.

**Figure 2:**
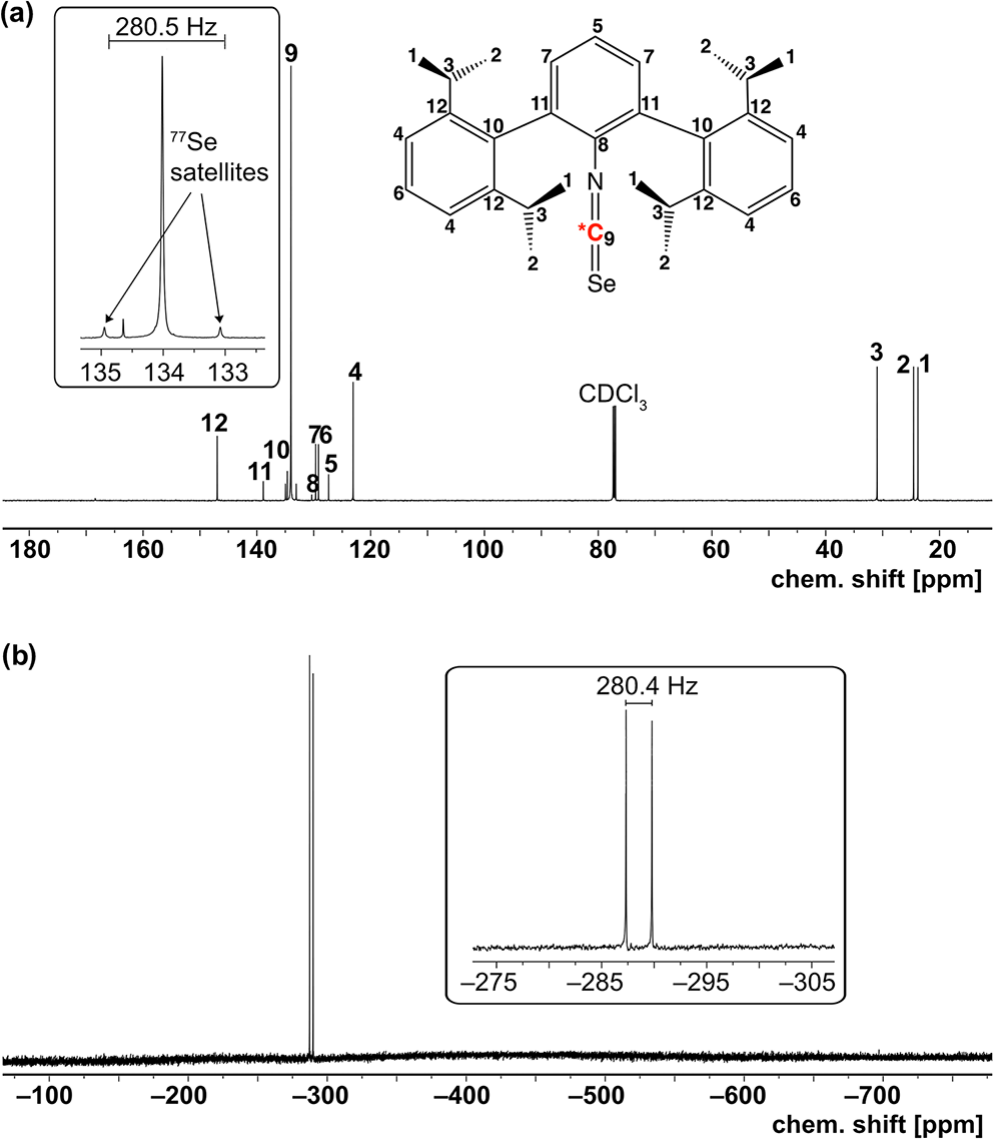
(a) ^13^C­{^1^H} and (b) ^77^Se NMR spectra of ^13^C-enriched Se*CNAr^Dipp2^ in CDCl_3_.

Another remarkable feature of the recorded ^77^Se NMR spectra is the clear dependence of the chemical shifts on the solvents used. Interestingly, there are almost no variations in nonpolar solvents such as CDCl_3_ and toluene, while a considerable upfield-shift of the signals is observed for all measured samples when the spectra were recorded in tetrahydrofuran. Unfortunately, similar spectra could not be recorded in other donor solvents such as alcohols, DMSO, or acetone due to decomposition of the samples. The chemical shifts observed in CDCl_3_ and THF are compared in Figure 3, where also some literature values have been included. The corresponding values for some of the isoselenocyanates obtained in toluene can be found in Experimental Section.

**Figure 3:**
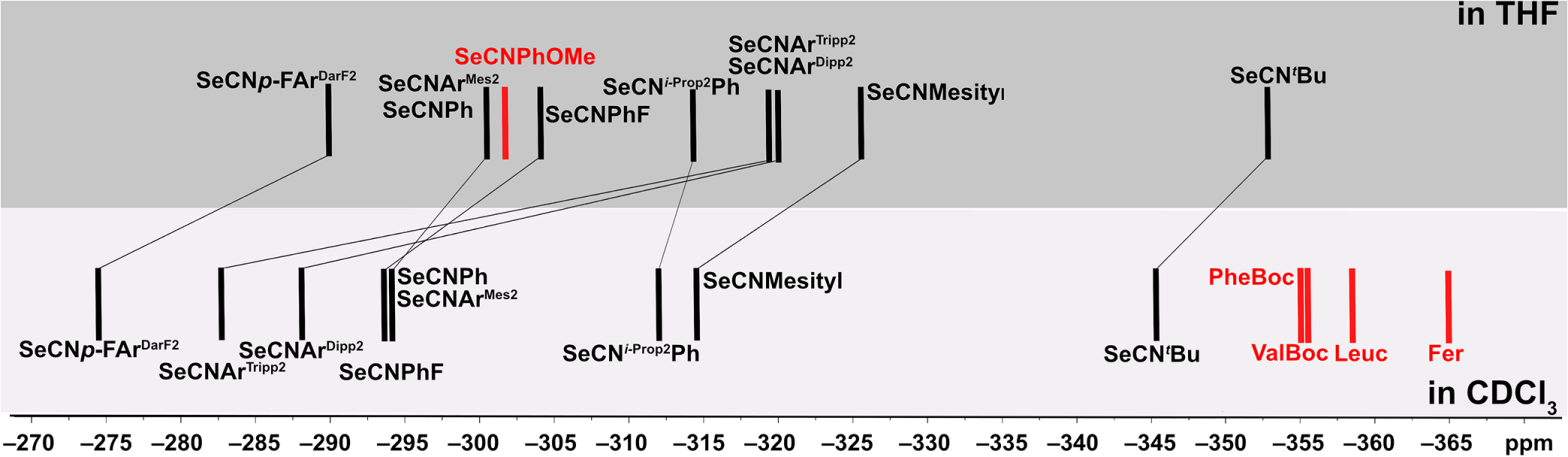
^77^Se NMR chemical shifts of isoselenocyanates in CDCl_3_ and THF. The values refer to the compounds of the present study and some literature data in red color (PheBoc: Boc-Phe-Ψ­[CH_2_NCSe],^22^ ValBoc: Boc-Val-Ψ­[CH_2_NCSe],^22^ Leuc: *Z*-Leu-Ψ­[CH_2_NCSe],^22^ Fer: 1,1-diisoselenocyanatoferrocene).^23^

Irrespective of the organic residues, the signals in THF indicate a significantly higher shielding of the selenium nuclei, ranging from 3.7 ppm (SeCN^*i*‑Prop2^Ph) to 35.5 ppm (SeCNAr^Tripp2^). Another systematic study on solvent effects in ^77^Se NMR spectroscopy has been conducted on a series of eight different diphenyl diselenides, where a similar trend between measurements in several nonpolar and polar solvents (benzene, chloroform, methanol, acetic acid, THF, pyridine, acetonitrile, DMSO) was found.^35^ The reported solvent-dependent differences of the chemical shifts, however, are clearly smaller and range between 3 and typically less than 10 ppm, with the exception of the values in DMSO, where upfield shifts up to 20 ppm with respect to CDCl_3_ have been found. As one of the possible reasons for such findings, variations in the dipole moments of the solvents have been attributed. However, it also became clear that other effects, e.g., due to the electronic properties should have an influence.^35^ This is also evident for the isoselenocyanates of the present study, where the variations in the chemical shifts due to the organic residues and the solvents are almost of the same magnitude. It would be interesting to attribute the experimental data to individual effects such as the electronic properties of the organic residues, steric factors or noticeable interactions between the {SeCN}^−^ unit with the solvent molecules. With this in mind, and given our long-term interest in noncovalent interactions of heavier chalcogen atoms,^49–51^ we decided to address these points by some DFT calculations on the B3LYP/def2tzvp level. Despite significant progress in the recent years,^52–56^ the accurate computation of ^77^Se chemical shifts remains a challenge. A complicated interplay of several factors (relativistic and vibrational effects, influences of solvents, electronic or steric factors of organic residues) emerges,^53^ which makes it difficult to derive one appropriate parameter set for the simulation of accurate chemical shifts of a larger variety of selenium compounds belonging to the same family, but having different residues. Thus, commonly deviations of a few tens of ppm between experimental and calculated are observed when solvent effects can be minimized.^52–54^ This is also the case for the isoselenocyanates of the present study, where the theoretically predicted gas-phase GIAO chemical shifts at the B3LYP/*x*2c-TZVPPall-s correlate with the observed chemical shifts in CHCl_3_ with a mean absolute error of 36 ppm (*R* ^2^ ≈ 0.7). A comparison of the experimental data with the results of the gas-phase calculations and with such obtained with the application of an implicit solvent model calculation (integral equation formalism polarizable continuum model; IEF-PCM) at the gas-phase geometries is given as tabular material and in graphical form in the Supporting Information of this paper. Interestingly, the experimental trend for a more positive chemical shift in a donor solvent is already well-reproduced, with a mean absolute error of 3.0 ppm *in silico* by a simple implicit solvation approach. Thus, solvent interactions must be subtle, and it became interesting to also consider the explicit association of a donor-solvent molecule such as THF with the model isoselenocyanates to better understand the observed effects.

A careful inspection of the electrostatic potential (ESP) at the surface of some model isoselenocyanate compounds gives evidence for the appearance of regions with positive electrostatic potential (σ-holes) at the back side of the C–Se bonds. Their magnitude depends on the organic residues of the isoselenocyanates. While alkyl substituents and aryl substituents with electron-donating groups such as OMe in *para* position show a weak σ-hole, electron-withdrawing substituents such as F^–^ (e.g., in SeCN*p*-FAr^DArF2^) cause a significant increase in the observed σ-hole at the back side of the C–Se bond. This is shown as an example in Figure 4 for SeCN^*t*^Bu and SeCN*p*-FAr^DArF2^. More examples given as Supporting Information.

**Figure 4:**
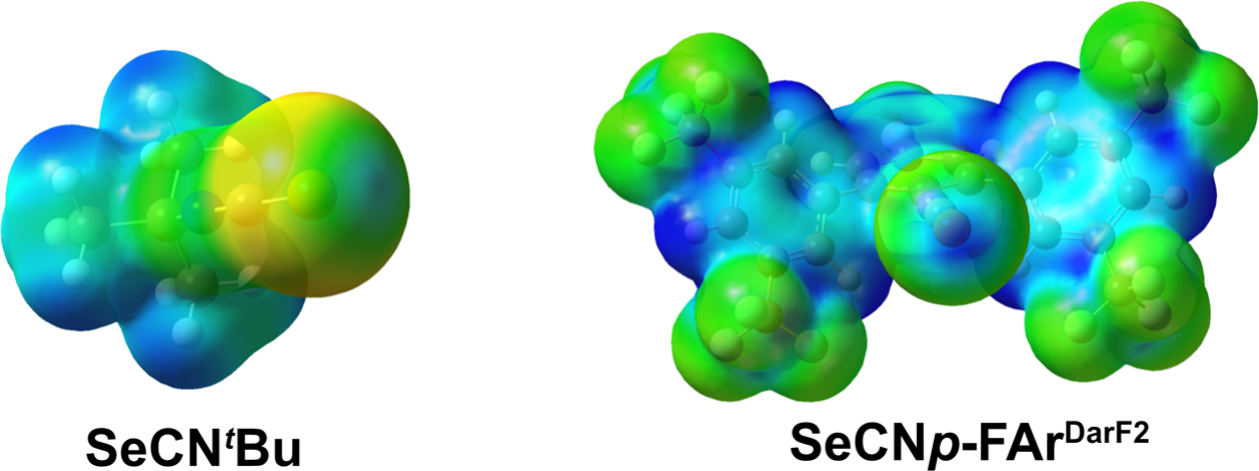
Molecular electrostatic potential mapping of SeCN^*t*^Bu and SeCN*p*-FAr^DArF2^ (depicted color scaled to the boundary surface potential maxima of SeCN^*t*^Bu) at an isosurface electron density level of 0.004 e/A^3^.

The established σ-holes are clearly too weak to form stable aggregates with weakly coordinating lone pairs of the chlorine atoms in CH_2_Cl_2_ or CHCl_3_. However, a weak, directional interaction with the oxygen atom of THF was identified in topological, interaction region indicator (IRI) and reduced density gradient (RDG) analyses. The corresponding illustrations are given in the Supporting Information. The related energies are consistent with van der Waals interactions and relatively insensitive to changes in the electronic situation at the isoselenocyanates judging from nearly indistinguishable RDG features between the electronically diverse model series of compounds. It shall be mentioned that NMR calculations performed for the explicit THF adducts show larger mean absolute deviations for the relative solvent shift compared to the implicit solvation models, likely due to an overevaluation of the explicit interaction energy. Thus, we attribute the chemical shift changes to changes in the internal resonance structure of the isoselenocyanates (i.e., favoring a zwitterionic resonance formula (R)­N^+^≡C–Se^–^ over the charge-neutral formulation (R)­N=C=Se or *vice versa*, depending on the solvent polarity) rather than explicit interactions with the σ-hole.

To gain more insights into the structural chemistry of the isoselenocyanates in the solid state, we determined the molecular structures of a series of the SeCNR compounds by single-crystal X-ray diffraction. Figure 5 illustrates the molecular structures of SeCN^*t*^Bu and SeCNMesityl, isoselenocyanates with the smallest steric bulk. Already at first glance, it becomes clear that the isocyanate units in these both compounds are freely accessible and no steric restrictions are visible (illustrations of space-filling models are contained as Supporting Information).

**Figure 5:**
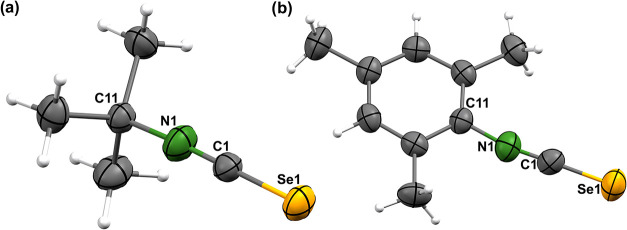
Ellipsoid representations of the molecular structures of (a) SeCN^*t*^Bu and (b) SeCNMesityl. Thermal ellipsoids represent 50% probability.

A completely different situation is found for the *m*-terphenyl isoselenocyanates, where the space around the {SeCN}^−^ groups is widely occupied by the presence of organic residues. The molecular structures of these compounds are depicted in Figure 6. In addition to their ellipsoidal representations, space-filling models are shown. They illustrate the different degrees of steric shielding in the individual compounds.

**Figure 6:**
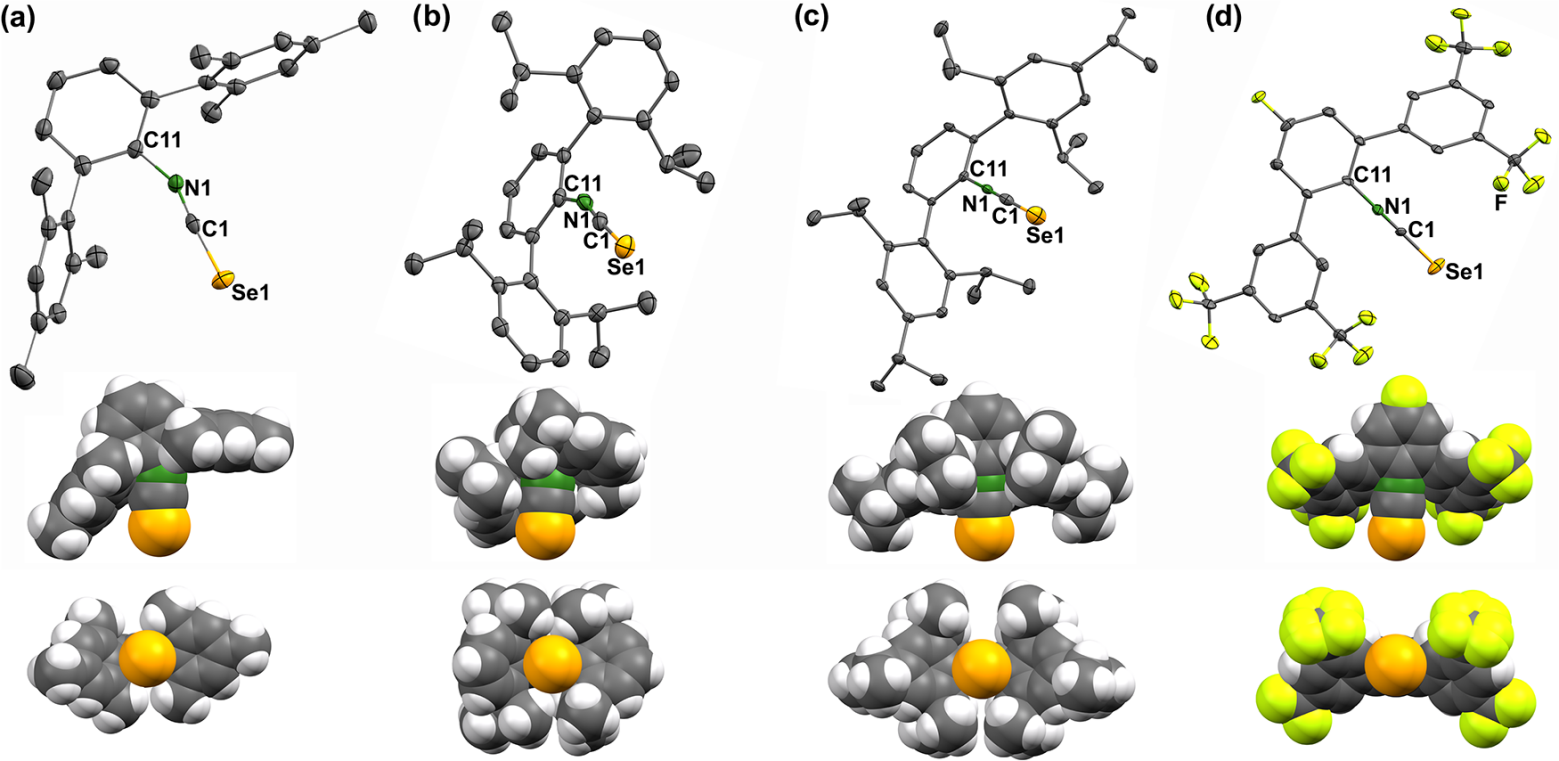
Ellipsoid representations and each two space-filling models of the molecular structures of (a) SeCNAr^Mes2^, (b) SeCNAr^Dipp2^, (c) SeCNAr^Tripp2^, and (d) SeCN*p*-FAr^DarF2^. Thermal ellipsoids represent 50% probability and the hydrogen atoms in the corresponding illustrations are omitted for clarity. The space-filling models are drawn on the basis of the van der Waals radii.

The nitrogen atoms are fully embedded in molecular frameworks of all regarded *m*-terphenyl isoselenocyanates. But interestingly, a full (almost spherical) protection of the selenium and carbon atoms of the isoselenocyanate groups is only given in SeCNAr^Tripp2^, while these molecular positions remain accessible for ongoing reactions in SeCNAr^Mes2^, SeCNAr^Dipp2^, and SeCN*p*-FAr^DarF2^. This is nicely illustrated by the top views (along the SeCN axes) of the space-filling models in Figure 6 and underlines the unique role of {Ar^Tripp2^} residues by providing the highest degree of steric protection in this family of encumbered substituents. Indeed, the corresponding isocyanide has previously been used for the protection of several metal ions with unusual metal centers and sensitive ligands.^57–62^ Finally, the high steric protection of two CNAr^Tripp2^ ligands allowed the isolation of the unique iron complex [Fe­(BF)­(CO)_2_(CNAr^Tripp2^)] that has a unique linear BF ligand in the coordination sphere of the transition metal.^63^

Table 1 contains selected bond lengths and angles of the novel isoselenocyanates. The dimensions of the Se–C and C–N bonds are unexceptional and in the range of previously observed values.^22–27^ They reflect the expected π-bonding within these units, and consequently, the isoselenocyanate groups are linear with Se1–C1–N1 angles between 175.5(2) and 180.0(5)°. A relatively wide variance is observed for the C11–N1–C1 angles with values between 154.9(2)° and 179.7(5)°, which is most probably due to crystallographic packing effects or the steric bulk of the organic substituents. The role of the latter influence is supported by the related angles in the *m*-terphenyl isoselenocyanates of Figure 5. The lowest C11–N1–C1 angles are observed for SeCNAr^Mes2^ and SeCNAr^Dipp2^, where the steric stress from the organic residues can be minimized by bending the {SeCN}^−^ units in the opposite direction. The more bulky residues in SeCN*p*-FAr^DarF2^ and particularly in SeCNAr^Tripp2^ provide a “more spherical” shielding around the {SeCN}^−^ groups and, thus, do not allow minimization of steric strains by a significant modification of the corresponding bonding angles. It should be mentioned that in phenyl isoselenocyanates the {SeCN}^−^ substituents are coplanar with the central phenyl rings, which supports an extended π-bonding.

**Table 1:** Selected Bond Lengths (Å) and Angles (°) in the Isoselenocyanates

	Se–C1	C1–N1	N1–C11	Se–C1–N1	C1–N1–C11
SeCN^*t*^Bu	1.741(5)	1.162(6)	1.450(6)	180.0(5)	175.9(5)
SeCNMesityl	1.720(5)	1.176(6)	1.399(6)	177.9(5)	168.8(5)
SeCNAr^Mes2^	1.726(3)	1.184(3)	1.387(3)	175.5(2)	154.9(2)
SeCNAr^Dipp2^	1.744(4)	1.158(5)	1.402(5)	176.4(4)	156.7(4)
SeCNAr^Tripp2^	1.741(4)	1.154(5)	1.398(5)	179.4(4)	179.7(5)
SeCN*p*-FAr^DarF2^	1.716(12)	1.143(17)	1.398(19)	179.8(12)	172.3(14)

Reactions of isocyanides with elemental selenium represent a facile approach to organoisoselenocyanates and can be used for aliphatic and aromatic substrates. The addition of a supporting base is required to avoid the immediate decomposition of the formed products and its crucial role for the formed products has been described previously by Mohr et al.^27^ Judging from the theoretical investigations of donor-solvent interactions with the isoselenocyanates (vide supra), an interaction of the base with the weak σ-hole at selenium could support the stabilization of the isoselenocyanates *in situ* beyond the quenching of hydrolytically released acid upon inadvertent hydrolysis with traces of water. DFT calculations at the B3LYP/def2tzvp level (for details, see Supporting Information) reveal a weak van der Waals interaction similar to that found for the solvent THF discussed above between the lone pair of NEt_3_ and the selenium atom. An alternative mechanistic involvement of the base was roughly checked; however, the conceivable catalytic selenium oxidation of the CN triple bond via the activation of elemental selenium by a nitrogen­(V) species, SeNR_3_, is energetically unlikely. The gas-phase reaction of free isocyanides R-NC with Se_8_ to form R-NCSe is energetically favored with approximately 30 kJ/mol for aliphatic isocyanides and approximately 40 kJ/mol for aromatic isocyanides. In principle, and in accordance with these results, the added base should only be required to capture hydrolytically released protons and avoid acid-catalyzed polymerization. However, the use of tertiary amines such as triethylamine is recommended to suppress undesired side-reactions, such as the formation of selenoureas. Similar findings have been reported occasionally before.^21,27,34,64–66^

In one example, we used the secondary amine HN­(*i*-Prop)_2_, the basicity of which is comparable to that of the less nucleophilic base NEt_3_, for a reaction between CN*p*-FAr^DarF2^ and selenium (Scheme 2). The main product was again the corresponding isoselenocyanate, but the yield was somewhat lower than that with NEt_3_. Unlike during the reactions with triethylamine, an additional nucleophilic attack on the isoselenocyanate carbon atom by the secondary amine and the formation of a selenourea was observed.

**Scheme 2:**
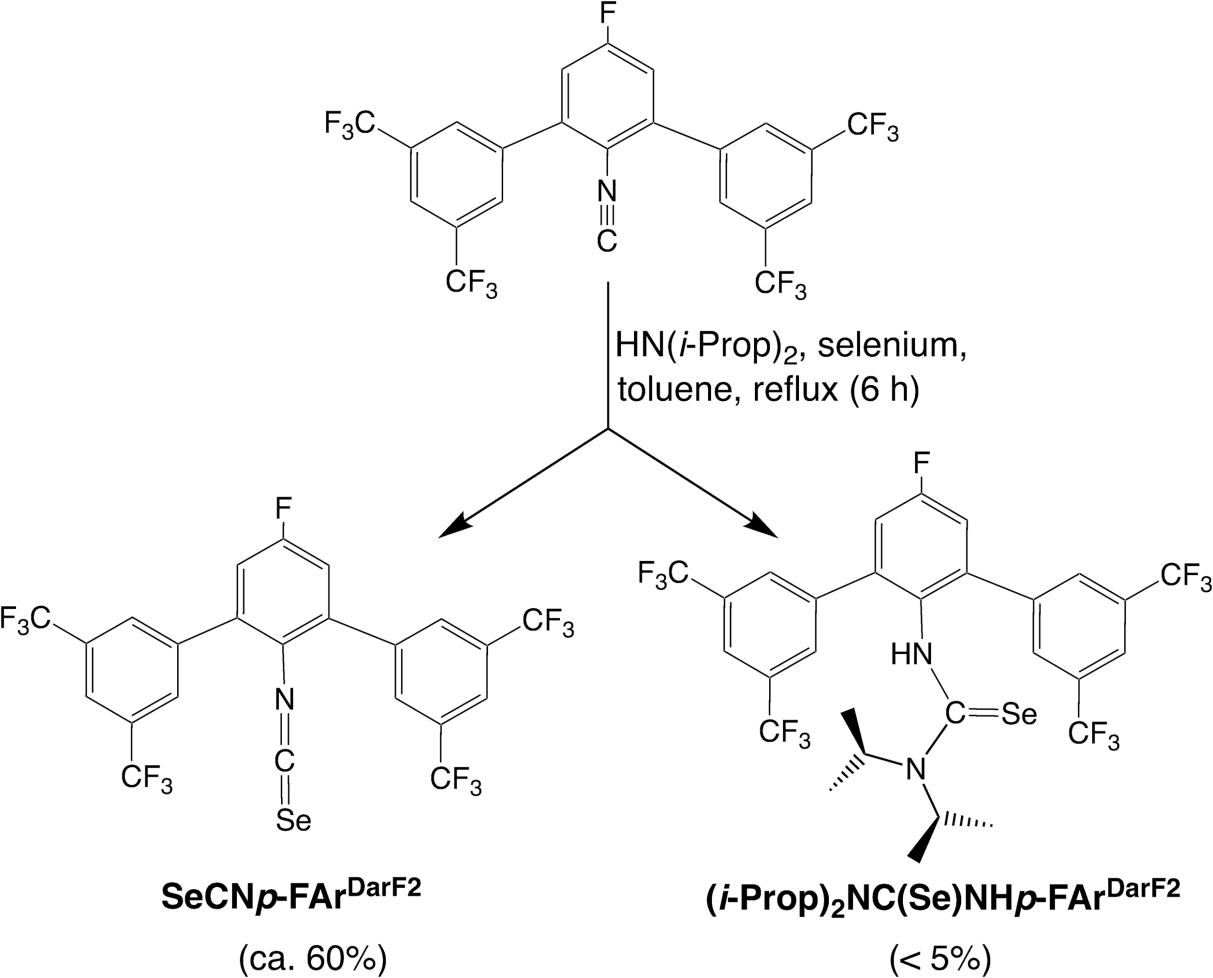
Reactions between CN*p*-FAr^DarF2^ and Elemental Selenium with HN­(*i*-Prop)_2_ as a Supporting Base

The suggested reaction pathway by a follow-up reaction is energetically feasible at elevated temperatures, according to DFT calculations at the B3LYP/def2tzvp level. However, the product is significantly less stable compared to a free isoselenocyanate and HN­(*i*-Prop)_2_ in the gas-phase and in the THF solution (ΔΔ*G*_gas_ = 30 kJ/mol; ΔΔ*G*_THF_ = 20 kJ/mol). A small amount of this compound was isolated in crystalline form, and its structure was elucidated by single-crystal X-ray diffraction. An ellipsoid representation of the structure of the product is shown in Figure 7.

**Figure 7:**
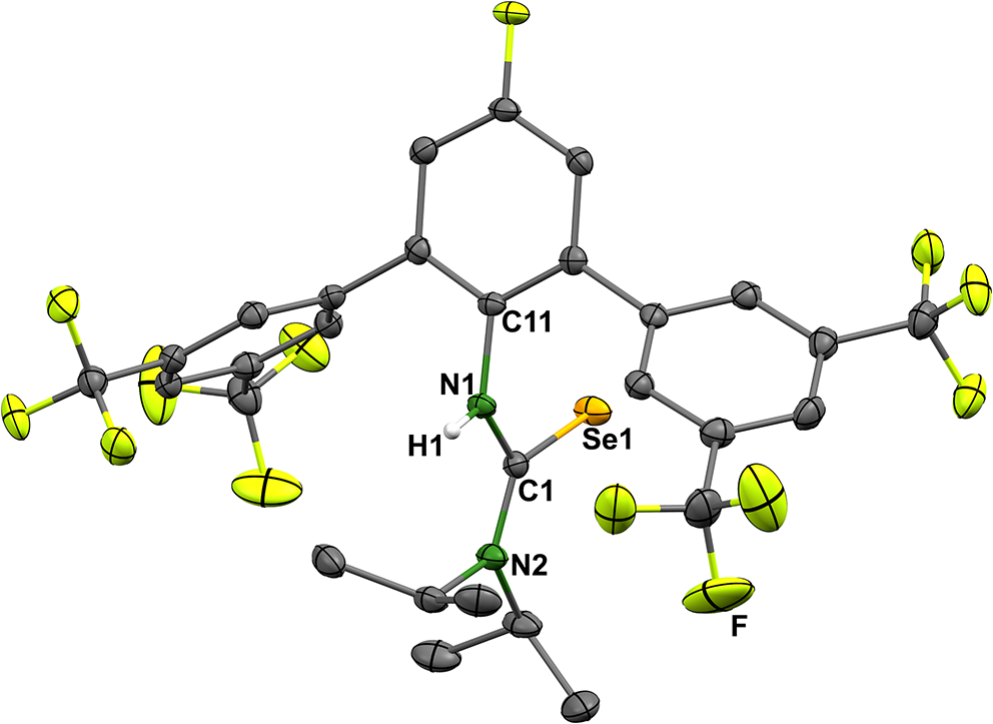
Ellipsoid representations of the structure of (*i*-Prop)_2_NC­(Se)­NH*p*-FAr^DarF2^. Thermal ellipsoids represent 50% probability. Hydrogen atoms bonded to carbon atoms are omitted for clarity. Selected bond lengths and angles: Se1–C1 1.859(2) Å, C1–N1 1.354(2) Å, C1–N2 1.337(2) Å, N1–C11 1.432(2) Å, N1–C1–N2 118.0(2)°, N1–C1–Se1 117.2(1)°, N2–C1–Se1 124.9(1)°.

The formation of selenoureas in reactions of isocyanides with selenium and amines has occasionally been reported.^27,64–66^ Such reactions found also consideration for tracer syntheses of such radioactive ^75^Se compounds.^67^ In the light of the promising results reported in the early paper of *Lipp* and co-workers^64^ and the ready formation of (*i*-Prop)_2_NC­(Se)­NH*p*-FAr^DarF2^ as an unintended side-product during the synthesis of SeNC*p*-FAr^DarF2^, this synthetic approach to substituted selenoureas seems to be promising. This, however, is beyond the scope of the present communication.

## Experimental Section

### Materials

All chemicals used in this study were of reagent grade and used without further purification. Solvents were dried and used as freshly distilled unless otherwise stated. The isocyanides were supplied commercially (CN^*t*^Bu) or prepared following previously published procedures (CNAr^Mes2^,^68^ CNAr^Dipp2^,^69^ CNAr^Tripp2^,^70^ CN*p*-FAr^DarF2^,^70^ CNPh,^71^ CNMesityl,^71^ CN^*i*‑Prop2^Ph,^71^ CNPhF^71^).

### Physical Measurements

Infrared spectra were measured on a Thermo Scientific Nicolet iS10 ATR spectrometer. NMR spectra were taken with JEOL 400 MHz or Bruker 600 MHz multinuclear spectrometers. Elemental analysis of carbon, hydrogen, and nitrogen were determined using a Heraeus vario EL elemental analyzer.

### Syntheses

The isoselenocyanates in this study were prepared by reactions of elemental selenium with a slight excess of the corresponding isocyanides and an appropriate base in dry solvents under an inert atmosphere. The solids deposited during slow concentration of the solutions in a vacuum. The oily products were isolated by the removal of the volatiles in vacuum. Individual reaction conditions, solvents, yields, and analytical data are given below.

#### SeCN^t^Bu

1.74 g (21 mmol) of CN^*t*^Bu, 1.18 g (15 mmol) of Se, 1.52 g (15 mmol) NEt_3_, solvent: THF, reaction time: 6 h. Yield: 90%. Colorless solid. IR (ATR, cm^–1^): 2981(s), 2936­(w), 2868­(w), 2431­(w), 2129­(vs) (ν_CN_), 2096(s) (ν_CN_), 1780­(w), 1490­(m), 1365­(vs), 1232(s), 1194­(vs), 1167(s), 1140­(w), 930­(w), 770­(m), 720­(w). ^1^H NMR (CDCl_3_, ppm): 1.15 (s, CH_3_). ^77^Se NMR (ppm, solvent): −345.5 (CDCl_3_), −353.3 (THF).

#### SeCNPh

540 mg (5.25 mmol) of CNPh, 295 mg (3.75 mmol) of Se, and 380 mg (3.75 mmol of NEt_3_), solvent: THF, reaction time: 3 h. Yield: 75%. Reddish oil. IR (ATR, cm^–1^): 3057(s), 2112­(vs) (ν_CN_), 2045(s) (ν_CN_), 2046­(w), 1587(s), 1492(s), 1448­(w), 1381­(m), 1321­(m), 1266­(m), 1206­(m), 1167­(m), 1155­(w), 1071­(m), 1025­(m), 904­(w), 844­(w), 751(s), 689­(vs), 613­(m). ^77^Se NMR (ppm, solvent): −294.6 (CDCl_3_), −301.2 (THF). ^1^H and ^13^C NMR spectra and elemental analyses of sufficient quality could not be measured due to an ongoing decomposition of the compound.

#### SeCNMesityl

380 mg (2.63 mmol) of CNMesityl, 150 mg (1.88 mmol) of Se, 190 mg (1.88 mmol NEt_3_), solvent: THF, reaction time: 3.5 h. Yield: 70%. Colorless solid. IR (ATR, cm^–1^): 2974­(w), 2910­(m), 2851­(w), 2108­(vs) (ν_CN_), 2018­(m) (ν_CN_), 1743­(w), 1679­(m), 1669­(m), 1581­(w), 1523­(w), 1473­(m), 1451(s), 1436­(m), 1380­(m), 1373­(w), 1353­(w), 1309­(m), 1261­(m), 1216­(w), 1177­(m), 1094­(w), 1032(s), 957­(w), 890­(w), 853­(vs), 804­(vs), 713(s), 702­(m), 592­(w), 562­(m). ^1^H NMR (CDCl_3_, ppm): 6.76 (s, 2H, phenyl), 2.41 (s, 6H, CH_3_), 2.41 (s, 3H, CH_3_). ^77^Se NMR (ppm, solvent): −314.4 (CDCl_3_), −325.3 (THF).

#### SeCN^i‑Prop2^Ph

490 mg (2.63 mmol) of CN^*i*‑Prop2^Ph, 150 mg (1.88 mmol) of Se, and 190 mg (1.88 mmol of NEt_3_), reaction time: 6 h. Yield: 80%. Colorless oil. IR (ATR, cm^–1^): 2963(s), 2929­(m), 2871­(m), 2110­(vs) (ν_CN_), 2090­(vs) (ν_CN_), 1686­(w), 1587­(w), 1460(s), 1434­(m), 1386­(m), 1364­(m), 1333­(w), 1259­(m), 1181­(m), 1165­(w), 1109­(m), 1044­(w), 849­(m), 794(s), 746­(vs), 691­(m), 626­(w), 577­(m). ^77^Se NMR (ppm, solvent): −311.2 (CDCl_3_), −314.9 (THF). ^1^H and ^13^C NMR spectra and elemental analyses of sufficient quality could not be measured due to an ongoing decomposition of the compound.

#### SeCNAr^Mes2^

360 mg (1.05 mmol) of CNAr^Mes2^, 59 mg (0.75 mmol) of Se, 76 mg (0.75 mmol of NEt_3_), solvent: toluene, reaction time: 6 h. Yield: 50%. Colorless solid. Elemental analysis: calcd for C_25_H_25_NSe: C, 71.8; H, 6.0; N, 3.4%. Found: C, 72.3; H, 5.8; N, 3.3%. IR (ATR, cm^–1^): 2967­(w), 2942­(w), 2913­(m), 2853­(w), 2086­(vs) (ν_CN_), 2040­(vs) (ν_CN_), 1612­(m), 1575­(m), 1438­(m), 1414­(m), 1375­(w), 1260­(m), 1094­(m), 1013­(m), 970­(w), 847(s), 780­(vs), 785(s), 764­(vs), 751(s), 739­(m), 666­(m), 601­(m), 580(s), 569­(m). ^1^H NMR CDCl_3_, ppm: 7.45 (t, 1H, p-phenyl), 7.20 (s, 4H, m-Mes), 7.00 (s, 2H, m-phenyl), 2.37 (s, 6H, p-CH_3_), 2.06 (s, 12H, o-CH_3_) ppm. ^13^C­{^1^H} NMR (CDCl_3_, ppm): 139.2, 137.8, 136.0, 134.4, 133.2 (NCSe), 129.5, 128.4, 128.1, 21.3 (p-CH_3_), 20.3 (o-CH_3_) ppm. ^77^Se NMR (ppm, solvent): −294.3 (CDCl_3_), −293.5 (toluene), −301.3 (THF).

#### SeCNAr^Dipp2^

Twenty-two milligrams (0.052 mmol) of CNAr^Dipp2^, 8 mg (0.1 mmol) of Se, 10 mg (0.10 mmol of NEt_3_), solvent: toluene, reaction time: 6 h. Yield: practically quantitative. Colorless solid. Elemental analysis: calcd for C_31_H_37_NSe: C, 74.1; H, 7.4; N, 2.8%. Found: C, 73.8; H, 7.4; N, 2.9%. IR (ATR, cm^–1^): 3064­(w), 2964(s), 2928­(m), 2887­(w), 2111(s) (ν_CN_), 2024(s) (ν_CN_), 1579­(w), 1462­(m), 1416­(w), 1375­(w), 1363­(w), 1251­(w), 1180­(w), 1057­(w), 806­(m), 791­(m), 584­(w), 501­(w). ^1^H NMR (CDCl3, ppm): 7.35 (t, 1H, *J* = 8 Hz, p-phenyl), 7.33 (t, 2H, *J* = 7 Hz, p-Dipp), 7.18 (d, 2H, *J* = 7 Hz, m-phenyl), 7.16 (d, 4H, *J* = 5 Hz, m-Dipp), 2.49 (sept, 4H, J = 5 Hz, CH, i-prop), 1.07 (d, 12H, *J* = 7 Hz, C­(CH3)­2, i-prop). ^13^C­{^1^H} NMR (CDCl_3_, ppm): 147.0, 138.9, 134.6, 134.0 (NCSe), 130.4, 129.7, 129.2, 127.4, 123.8, 31.0 (CH, i-prop), 24.6 (CH3, i-prop), 23.8 (CH3, i-prop). ^77^Se NMR (ppm, solvent): −286.5 (CDCl_3_), −320.0 (THF).

#### SeCNAr^Tripp2^

530 mg (1.05 mmol) of CNAr^Tripp2^, 59 mg (0.75 mmol) of Se, 76 mg (0.75 mmol of NEt_3_), solvent: toluene, reaction time: 6 h. Yield: 50%. Colorless solid. Elemental analysis: calcd for C_37_H_49_NSe: C, 75.7; H, 8.4; N, 2.4%. Found: C, 76.0; H, 8.5; N, 2.3%. IR (ATR, cm^–1^): 2955­(vs), 2924­(m), 2865­(m), 2121­(vs) (ν_CN_), 2063­(m) (ν_CN_), 1608­(w),1571­(w), 1458(s), 1428­(w), 1417­(m), 1381­(m), 1362(s), 1339­(w), 1314­(m), 1240­(w), 1172­(w), 1104­(m), 1069­(m), 1053­(m), 941­(m), 876(s), 854­(m), 800­(m), 774(s), 693­(m), 650­(m), 624­(w), 591­(w), 542­(w). ^1^H NMR (CDCl_3_, ppm): 7.35 (t, 1H, p-Ph), 7.25 (d, 2H, m-Ph), 7.05 (s, 4H, m-Tripp), 2.95 septet, 2H, p-(CH­(i-prop)_2_), 2.60 septet, 4H, o-(CH­(i-prop)_2_), 1.30 (d, 12H, CH_3_(i-prop)_2_), 1.18 (d, 24H, CH_3_(i-prop)_2_). ^13^C­{^1^H} NMR (CDCl_3_, ppm): 149.4, 146.6, 139.1, 135.0 (NCSe), 132.4, 131.2, 129.7, 127.1, 121.1, 34.6 (CH­(i-prop)_2_), 30.9 (CH­(i-prop)_2_), 24.6 (CH_3_(i-prop)_2_), 24.3 (CH_3_(i-prop)_2_), 24.2 (CH_3_(i-prop)_2_) 23.8 (CH_3_(i-prop)_2_). ^77^Se NMR (ppm, solvent): −283.8 (CDCl_3_), −286.1 (toluene), −319.2 (THF).

#### SeCNp-FAr^DarF2^

110 mg (0.21 mmol) of CN*p*-FAr^DarF2^, 33 mg (0.42 mmol) of Se, 42 mg (0.42 mmol NEt_3_), solvent: toluene, reaction time: 6 h. Yield: 99%. Light yellow solid. Elemental analysis: calcd for C_23_H_8_F_13_NSe: C, 44.3; H, 1.3; N, 2.2%. Found: C, 44.0; H, 1.3; N, 2.1%. IR (ATR, cm^–1^): 3091­(w), 2119(s) (νCN), 2051­(w) (νCN), 1827­(w), 1698­(w), 1621­(w), 1595­(m), 1474­(m), 1463­(m), 1416­(m), 1400(s), 1273­(vs), 1221­(m), 1169(s), 1125­(vs), 1109­(vs), 1066­(m), 969­(w), 917­(m), 906(s), 873­(m), 847­(m), 769­(w), 757­(w), 736­(m), 748­(m), 681(s), 634­(m), 616­(w), 585­(m). ^1^H NMR (CDCl_3_, ppm): δ = 7.94 (s, 2H, p-phenylF), 7.90 (s, 4H, o-phenylF), 7.19 (d, ^3^JHF = 5 Ht 2H, m-phenyl) ppm. ^13^C­{1H} NMR (CDCl_3_, ppm): 160.8 (d, ^1^JCF = 211 Hz), p-phenylF, 139.4 (d, ^3^JCF = 6 Hz, o-phenylF), 138.3, 134.5 (NCSe), 132.8 (q, ^2^JCF = 30 Hz, m-ArF), 129.4, 123.4 q, ^3^JCF = 3 Hz, 123.1 (q, ^1^JCF = 226 Hz, CF_3_), 123.1 (quintet, ^3^JCF = 3 Hz, p-ArF), 120.4, 118.1 (d, ^2^JCF = 20 Hz, m-phenylF) ppm. ^19^F NMR (CDCl_3_, ppm): −63.8 (s, CF_3_), −110.5 (t, ^3^JFH = 5 Hz, p-phenylF). ^77^Se NMR (ppm, solvent): −274.1 (CDCl_3_), −276.4 (toluene), −289.9 (THF).

#### SeCNPhF

320 mg (2.6 mmol) of CNPhF, 150 mg (1.88 mmol) of Se, 190 mg (1.88 mmol NEt_3_), solvent: THF, reaction time: 3 h. Yield: 25% (impure product). Reddish oil. ^77^Se NMR (ppm, solvent): −294.3 (CDCl_3_), −304.6 (THF). IR, ^1^H, and ^13^C NMR spectra, and elemental analyses of sufficient quality could not be measured due to an ongoing decomposition of the compound.

### X-ray Crystallography

The intensities for the X-ray determinations were collected on STOE or Bruker instruments with Mo Kα radiation (λ = 0.71073 Å). Standard procedures were applied for data reduction and absorption correction.^72,73^ Structure solution and refinement were performed with SHELX included in the OLEX2 program package.^74–76^ Hydrogen atoms were calculated for idealized positions and treated with the “riding model” option of SHELXL unless otherwise stated. MERCURY was used to prepare the structure representations.^77^ More details about the data collections, the structure calculations, and ellipsoid representations of the crystal structures are provided in the Supporting Information. The structural data are deposited with the Cambridge Crystallographic Data Centre with the deposition numbers CCDC-2489198 ([SeCN^*t*^Bu]), CCDC-2489199 (SeCNMesityl), CCDC-2489200 (SeCNAr^Mes2^), CCDC-2489201 (SeCNAr^Dipp2^), CCDC-2489202 (SeCNAr^Tripp2^), CCDC-2489203 (SeCN*p*-FAr^DarF2^), CCDC-2489204 ((*i*-Prop)_2_NC­(Se)­NHAr^DarF2^), and CCDC-2489205 (SeCN*p*-FAr^DarF2^ x CN*p*-FAr^DarF2^).

### Computational Details

DFT calculations were performed on the high-performance computing systems of the Freie Universität Berlin ZEDAT (Curta) using the program package GAUSSIAN 16.^78,79^ The gas-phase geometry optimizations were performed using coordinates derived from the X-ray crystal structures using GAUSSVIEW.^80^ The calculations were performed with the hybrid density functional B3LYP.^81–83^ The triple-ζ relativistic pseudopotential def2-TZVP basis set was applied to all atoms.^84^ Frequency calculations after the optimizations confirmed the convergence, and no imaginary frequencies were obtained. Electrostatic potential maps were generated from GAUSSIAN cube files using GAUSSVIEW. Further analyses of the obtained wave functions were performed with the free multifunctional wave function analyzer Multiwfn.^85,86^ The reduced density gradient (RDG) method was used as implemented in Multiwfn and visualized using the VMD package.^87,88^

## Conclusions

Reactions of isocyanides with elemental selenium in the presence of NEt_3_ represent a suitable approach to isoselenocyanates and have been applied for a series of alkyl- and aryl-substituted starting materials including sterically highly encumbered *m*-terphenyl isocyanides. The products are obtained in moderate to good yields as crystalline (or in some cases oily) materials. The remarkable stability of the isoselenocyanates with sterically demanding substituents recommends the use of such compounds as synthons for organoselenium chemistry and may give extended opportunities, particularly for the syntheses of selenium-containing heterocycles.

The ^77^Se NMR signals of the isoselenocyanates appear between −270 and −370 ppm and show a marked dependence on the solvents. DFT calculations on the B3LYP/def2tzvp level account for the presence of σ-holes at the selenium atoms. They are weak for alkyl isoselenocyanates and aryl compounds with electron-donating substituents but become more significant for representatives with electron-withdrawing groups. Such areas might be able to establish at least weak interactions with donor solvents.

## Supplementary Material



## Notes

IR, ^1^H NMR, ^77^Se NMR, ^13^C (where available) NMR spectra, and computational details (PDF)
