# Supplementary material for: Isoselenocyanates with Sterically Encumbered Substituents—Synthesis, Structures, and Spectroscopic Properties
Source: Inorg Chem. 2026 Mar 16;65(12):6942–52. doi: 10.1021/acs.inorgchem.6c00462 (PMC13040519; doi:10.1021/acs.inorgchem.6c00462)
Supplement: Supplementary file 1 [file ic6c00462_si_001.pdf]

Supporting Information to the paper entitled:

Isoselenocyanates with Sterically Encumbered Substituents – Synthesis, Structures and Spectroscopic Properties

Vania D. Schwade,[1*] Michael L. Neville,[2] Guilhem Claude,[3] Maximilian Roca Jungfer,[4] Adelheid Hagenbach,[3] Ernesto Schulz Lang,[1] Joshua S. Figueroa,[2*] Ulrich Abram[3*]

[1] Department of Chemistry, Natural and Exact Sciences Centre, Federal University of Santa Maria, Avenida Roraima, n.1000, 97105-900 Santa Maria, RS, Brazil; E-Mail: vania.schwade@ufsm.br

[2] Department of Chemistry, University of California San Diego, 9500 Gilman Drive, MC 0358, La Jolla, California 92093, United States; E-Mail: jsfig@ucsd.edu

[3] Institute of Chemistry and Biochemistry, Freie Universität Berlin, Fabeckstr. 34/36, Berlin D-14195, Germany; E-Mail: ulrich.abram@fu-berlin.de

[4] Institute for Nuclear Waste Disposal (INE), Karlsruhe Institute of Technology, Hermann-von-Helmholtz-Platz 1, 76344 Eggenstein-Leopoldshafen, Germany

Table of content

Crystallographic data S5

Table S1. Crystal data and structure determination parameters. S5

Figure S1. a) Ellipsoid representation of SeCN^{t}Bu with atomic labelling scheme. The thermal ellipsoids are set at a 50% probability level. Symmetry operation: ‘ x,y,1/2–z. b) Unit cell plot. S8

Table S2. Bond lengths (Å) and angles (°) in SeCN^{t}Bu. Symmetry operation: [1] x,y,1/2–z. S8

Figure S2. a) Ellipsoid representation of SeCNMesityl with atomic labelling scheme. The thermal ellipsoids are set at a 50% probability level. b) Unit cell plot. S8

Table S3. Bond lengths (Å) and angles (°) in SeCNMesityl. S8

Figure S3. a) Ellipsoid representation of SeCNAr^{Mes2} with atomic labelling scheme. The thermal ellipsoids are set at a 50% probability level. b) Unit cell plot. S9

Table S4. Bond lengths (Å) and angles (°) in SeCNAr^{Mes2}. S9

Figure S4. a) Ellipsoid representation of SeCNAr^{Dipp2} with atomic labelling scheme. The thermal ellipsoids are set at a 50% probability level. b) Unit cell plot. S10

Table S5. Bond lengths (Å) and angles (°) in SeCNAr^{Dipp2}. S10

Figure S5. a) Ellipsoid representation of SeCNAr^{Tripp2} with atomic labelling scheme. The thermal ellipsoids are set at a 50% probability level. Symmetry operation: ‘ x,y,0.5–z. b) Unit cell plot. S11

Table S6. Bond lengths (Å) and angles (°) in SeCNAr^{Tripp2}. Symmetry operation: ‘ x,y,0.5–z. S11

Figure S6. a) Ellipsoid representation of SeCN*p*-FAr^{DarF2} with atomic labelling scheme. The thermal ellipsoids are set at a 50% probability level. Symmetry operation: ‘ x,–y,z. b) Unit cell plot. S12

Table S7. Bond lengths (Å) and angles (°) in SeCN*p*-FAr^{DarF2}. Symmetry operation: ‘ x,–y,z. S12

Figure S7. a) Ellipsoid representation of (*i*-Prop)$_2$NC(Se)NH*p*-FAr^{DarF2} with atomic labelling scheme. The thermal ellipsoids are set at a 50% probability level. b) Unit cell plot. S13

Table S8. Bond lengths (Å) and angles (°) in (*i*-Prop)$_2$NC(Se)NH**p**-FAr^{DarF2}. S13

Figure S8. a) Ellipsoid representation of SeCN*p*-FAr^{DarF2} x CN*p*-FAr^{DarF2} with atomic labelling scheme. The thermal ellipsoids are set at a 50% probability level. Symmetry operation: ‘ x,–y,z. b) Unit cell plot. S15

Table S9. Bond lengths (Å) and angles (°) in SeCN*p*-FAr^{DarF2} x CN*p*-FAr^{DarF2}. Symmetry operation: ‘ x,–y,z. S15

Selected spectroscopic data S18

Figure S9. IR spectra (ATR) of a) SeCN^{t}Bu and b) SeCNPh. S18

Figure S10. IR spectra (ATR) of a) SeCNMesityl and b) SeCN$^{i\text{-}prop2}$Ph. S18

Figure S11. IR spectra (ATR) of a) SeCNAr^{Mes2} and b) SeCNAr^{Dipp2}. S18

Figure S12. IR spectra (ATR) of a) SeCNAr^{Tripp2} and b) SeCN*p*-FAr^{DarF2}. S19

Figure S13. ^{77}Se NMR spectra of SeCN^{t}Bu in $CDCl_3$ (a) and THF-d_8 (b). S19

Figure S14. ^{77}Se NMR spectra of SeCNPh in $CDCl_3$ (a) and THF-d_8 (b). S19

Figure S15. ^{77}Se NMR spectra of SeCNMesityl in $CDCl_3$ (a) and THF-d_8 (b). S20

Figure S16. ^{77}Se NMR spectra of SeCN(2,6-diisoprop)phenyl in $CDCl_3$ (a) and THF-d_8 (b). S20

Figure S17. ^{77}Se NMR spectra of SeCNAr^{Mes2} in toluene-d_8 (a) and THF-d_8 (b). S20

Figure S18. ^{13}C NMR spectrum of SeCNAr^{Mes2} in $CDCl_3$. S21

Figure S19. ^{77}Se NMR spectra of SeCNAr^{Dipp2} in $CDCl_3$ (a) and THF-d_8 (b). S21

Figure S20. ^{77}Se NMR spectra of SeCNAr^{Tripp2} in $CDCl_3$ (a) THF-d_8 (b). S21

Figure S21. ^{13}C NMR spectrum of SeCNAr^{Tripp2} in $CDCl_3$. S22

Figure S22. ^{77}Se NMR spectra of SeCN*p*-FAr^{DarF2} in toluene (a) THF-d_8 (b). S22

Figure S23. ^{13}C NMR spectrum of SeCN*p*-FAr^{DarF2} in $CDCl_3$. S22

Figure S24. ^{77}Se NMR spectra of SeCNPhF in $CDCl_3$ (a) THF-d_8 (b). S23

Table S10. ^{77}Se NMR chemical shifts (ppm) and ν_{CN} IR frequencies (cm^{-1}) of the isoselenocyanates and the corresponding isocyanides. S23

Figure S25. ^{1}H NMR spectrum of ^{13}C-enriched Se*CNAr^{Dipp} in $CDCl_3$. S23

Figure S26. ^{13}C NMR spectrum of ^{13}C-enriched Se*CNAr^{Dipp} in $CDCl_3$. S24

Figure S27. ^{77}Se NMR spectrum of ^{13}C-enriched Se*CNAr^{Dipp} in $CDCl_3$. S24

Computational data S25

Figure S28. Molecular electrostatic potential mapping of some representative isoselenocyanates, Se_8, $SeMe_2$ and SeCO (depicted color scaled to the boundary surface potential maxima of SeCN^{t}Bu) at an isosurface electron density level of 0.004 e/A³. S25

Figure S29. Reduced-density gradient (RDG) analysis for the interaction of isoselenocyanates with THF. S26

Figure S30. Interaction region indicator (IRI) maps for the interaction of isoselenocyanates with THF. S27

Table S11. Experimental vs. calculated ^{77}Se NMR chemical shifts (ppm; relative to IEF-PCM solvated $SeMe_2$); PheBoc: Boc-Phe-*Ψ*[CH_2NCSe],[22] ValBoc: Boc-Val-*Ψ*[CH_2NCSe],[22] Leuc: *Z*-Leu-*Ψ*[CH_2NCSe],[22] Fer: 1,1-diisoselenocyanatoferrocene.[23] Based on gas-phase optimized geometries at B3LYP/def2tzvp level; GIAO NMR prediction at B3LYP/x2c-TZVPPall-s level. S28

Figure S31. Graphical representations of the experimental ^{77}Se chemical shifts in $CHCl_3$ and the values of the gas phase simulations contained in Table S11. a) In the common spectral range of ^{77}Se NMR spectroscopy and b) as zoom into the region of interest indicating the deviations of the simulated data. S29

Table S12. Experimental vs. calculated ^{77}Se NMR chemical shifts (ppm; relative to CN^{t}Bu); PheBoc: Boc-Phe-*Ψ*[CH_2NCSe],[22] ValBoc: Boc-Val-*Ψ*[CH_2NCSe],[22] Leuc: *Z*-Leu-*Ψ*[CH_2NCSe],[22] Fer: 1,1-diisoselenocyanatoferrocene.[23] Based on gas-phase optimized geometries at B3LYP/def2tzvp level; GIAO NMR prediction at B3LYP/x2c-TZVPPall-s level. MAE: mean absolute error; R²: correlation coefficient. S30

Table S13. Comparison between different DFT work-flows; experimental vs. calculated ^{77}Se NMR chemical shifts (ppm; relative to CN^{t}Bu); PheBoc: Boc-Phe-*Ψ*[CH_2NCSe],[22] ValBoc: Boc-Val-*Ψ*[CH_2NCSe],[22] Leuc: *Z*-Leu-*Ψ*[CH_2NCSe],[22] Fer: 1,1-diisoselenocyanatoferrocene.[23] Based on gas-phase optimized geometries at B3LYP/def2tzvp level; GIAO NMR prediction at B3LYP/x2c-TZVPPall-s level. MAE: mean absolute error; R²: correlation coefficient. S30

Table S15. Calculated free energies for diverse references and mechanistically interesting hypothetical molecules. S31

Table S16. Calculated free energies for the nucleophilic attack of HN(*i*-Prop)$_2$ at SeCN*p*-FAr^{DarF2} forming a selenourea. S31

Crystallographic data

Table S1. Crystal data and structure determination parameters.

	SeCN^{t}Bu	SeCNMesityl	SeCNAr^{Mes2}
Empirical formula	C_5H_9NSe	$C_{10}H_{11}NSe$	$C_{25}H_{25}NSe$
Formula weight	162.09	224.16	418.42
Temperature/K	293	293	273
Crystal system	orthorhombic	triclinic	orthorhombic
Space group	*Pbcm*	$P\bar{1}$	*Pbca*
a/Å	5.7216(11)	8.271(2)	9.0516(13)
b/Å	15.002(3)	8.257(3)	15.298(2)
c/Å	8.4253(17)	8.952(2)	30.448(4)
α/°	90	75.37(2)	90
β/°	90	72.36(2)	90
γ/°	90	59.95(2)	90
Volume/Å^3	723.2(2)	500.5(3)	4216.1(10)
Z	4	2	8
ρ_{calc} / g/cm^3	1.489	1.487	1.318
μ/mm^{-1}	5.085	3.697	1.790
F(000)	320.0	224.0	1728.0
Crystal size/mm^3	0.3 × 0.09 × 0.08	0.32 × 0.25 × 0.08	0.25 × 0.16 × 0.09
Radiation	MoKα (λ = 0.71073)	MoKα (λ = 0.71073)	MoKα (λ = 0.71073)
2Θ range for data collection/°	7.122 to 58.472	9.576 to 51.99	5.236 to 49
Index ranges	–7 ≤ h ≤ 7, –20 ≤ k ≤ 16, –11 ≤ l ≤ 11	–10 ≤ h ≤ 10, –8 ≤ k ≤ 10, –11 ≤ l ≤ 11	–10 ≤ h ≤ 10, –17 ≤ k ≤ 17, –34 ≤ l ≤ 35
Reflections collected	6342	4086	23967
Independent reflections	1036 [R_{int} = 0.0949, R_{sigma} = 0.0520]	1949 [R_{int} = 0.0856, R_{sigma} = 0.1097]	3499 [R_{int} = 0.1879, R_{sigma} = 0.0824]
Data/restraints/parameters	1036/0/49	1949/0/112	3499/0/250
Goodness-of-fit on F^2	1.032	0.822	1.033
Final R indexes [I>=2σ (I)]	R_1 = 0.0428, wR_2 = 0.1019	R_1 = 0.0451, wR_2 = 0.0878	R_1 = 0.0429, wR_2 = 0.1076
Final R indexes [all data]	R_1 = 0.0641, wR_2 = 0.1100	R_1 = 0.1115, wR_2 = 0.1047	R_1 = 0.0526, wR_2 = 0.1125
Largest diff. peak/hole / e Å^{-3}	0.79/–0.77	0.40/–0.45	0.52/–0.79
Diffractometer	STOE IPDS T2	STOE IPDS T2	Bruker CCD
CCDC access code	2489198	2489199	2489200

Table S1. Crystal data and structure determination parameters (continued).

	$SeCNAr^{Dipp2}$	$SeCNAr^{Tripp2}$	SeCN*p*-FAr^{DarF2}
Empirical formula	$C_{31}H_{37}NSe$	$C_{37}H_{49}NSe$	$C_{23}H_{8}F_{13}NSe$
Formula weight	502.57	585.73	624.26
Temperature/K	273	273	293
Crystal system	monoclinic	orthorhombic	monoclinic
Space group	$P2_1/n$	*Pbcm*	*Cm*
a/Å	9.9230(7)	10.9331(7)	8.9830(18)
b/Å	13.7669(11)	12.0503(7)	28.730(6)
c/Å	19.531315)	25.1456(14)	4.7014(9)
α/°	90	90	90
β/°	92.774(4)	90	106.38(3)
γ/°	90	90	90
Volume/$Å^3$	2665.0(4)	3312.9(3)	1164.1(4)
Z	4	4	2
ρ_{calc} / g/cm^3	1.253	1.176	1.781
μ/mm^{-1}	1.427	1.157	1.730
F(000)	1056	1248	608
Crystal size/mm^3	0.17 × 0.10 × 0.05	012 × 0.12 × 0.05	0.12 × 0.1 × 0.1
Radiation	Mo Kα (λ = 0.71073)	Mo Kα (λ = 0.71073)	Mo Kα (λ = 0.71073)
2Θ range for data collection/°	5.064 to 50.868	4.938 to 50.732	8.512 to 51.984
Index ranges	–11 ≤ h ≤ 11, –16 ≤ k ≤ 16, –23 ≤ l ≤ 23	–11 ≤ h ≤ 12, –14 ≤ k ≤ 12, –30 ≤ l ≤ 30	–11 ≤ h ≤ 11, –35 ≤ k ≤ 35, –5 ≤ l ≤ 5
Reflections collected	91742	11665	4816
Independent reflections	4900 [R_{int} = 0.2466, R_{sigma} = 0.0790]	3070 [R_{int} = 0.0829, R_{sigma} = 0.0731]	2090 [R_{int} = 0.1352, R_{sigma} = 0.0813]
Data/restraints/parameters	4900/0/306	3070/0/190	2090/221/209
Goodness-of-fit on F^2	1.019	1.041	1.025
Final R indexes [I>=2σ (I)]	R_1 = 0.0543, wR_2 = 0.1146	R_1 = 0.0503, wR_2 = 0.1080	R_1 = 0.0675, wR_2 = 0.1735
Final R indexes [all data]	R_1 = 0.1055, wR_2 = 0.1371	R_1 = 0.0767, wR_2 = 0.1214	R_1 = 0.0731, wR_2 = 0.1807
Largest diff. peak/hole / e $Å^{-3}$	0.67/–0.68	0.37/–0.56	0.48/–1.09
Diffractometer	Bruker CCD	Bruker CCD	STOE IPDS 2T
CCDC access code	2489201	2489202	2489203

Table S1. Crystal data and structure determination parameters (continued).

	(i-Prop)$_2$NC(Se)NHAr^{DarF2}	SeCNp-FAr^{DarF2} x CNp-FAr^{DarF2}
Empirical formula	$C_{29}H_{23}F_{13}N_2Se$	$C_{46}H_{16}F_{26}N_2Se$
Formula weight	725.45	1169.57
Temperature/K	273	293
Crystal system	monoclinic	monoclinic
Space group	$P2_1/c$	Cc
a/Å	12.1197(6)	29.830(5)
b/Å	19.9367(11)	9.156(2)
c/Å	12.5935(6)	17.040(2)
α/°	90	90
β/°	97.451(3)	95.860(10)
γ/°	90	90
Volume/Å^3	3017.2(3)	4629.7(14)
Z	4	4
ρ_{calc} / g/cm^3	1.597	1.678
μ/mm^{-1}	1.348	0.953
F(000)	1448	2296
Crystal size/mm^3	0.40 × 0.22 × 0.10	0.10 x 0.09 x 0.09
Radiation	Mo Kα (λ = 0.71073)	Mo Kα (λ = 0.71073)
2Θ range for data collection/°	4.842 to 50.872	4.656 to 49.998
Index ranges	$-14 \le h \le 14$, $-24 \le k \le 24$, $-15 \le l \le 15$	–35<=h<=34, –10<=k<=10, 0<=l<=20
Reflections collected	64229	7834
Independent reflections	5563 [R_{int} = 0.0469, R_{sigma} = 0.0202]	4096 [R_{int} = 0.0814, R_{sigma} = 0.0844)]
Data/restraints/parameters	5563/0/410	4095/4/647
Goodness-of-fit on F^2	1.039	1.027
Final R indexes [I>=2σ (I)]	R_1 = 0.0253, wR_2 = 0.0570	R_1 = 0.0569, wR_2 = 0.1426
Final R indexes [all data]	R_1 = 0.0303, wR_2 = 0.0591	R_1 = 0.066, wR_2 = 0.1446
Largest diff. peak/hole / e Å^{-3}	0.29/–0.38	1.30/–0.50
Diffractometer	Bruker CCD	Bruker CCD
CCDC access code	2489204	2489205

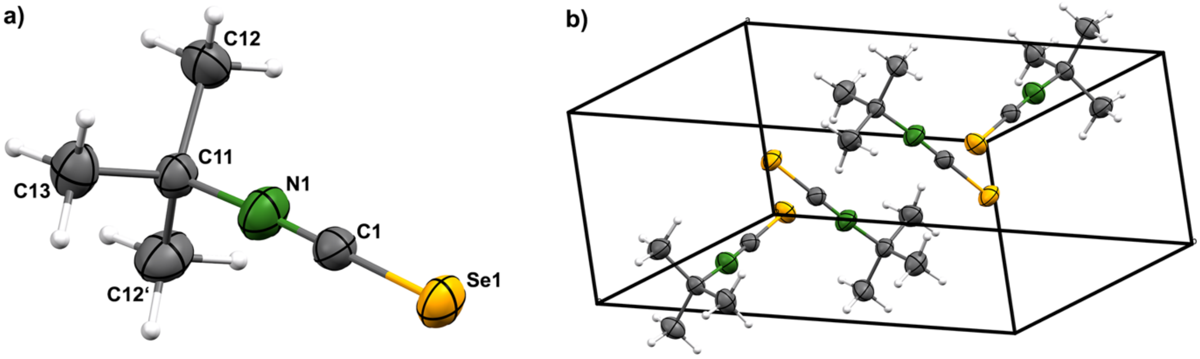

Figure S1. a) Ellipsoid representation of SeCN^{t}Bu with atomic labelling scheme. The thermal ellipsoids are set at a 50% probability level. Symmetry operation: ‘ x,y,1/2–z. b) Unit cell plot.

Table S2. Bond lengths (Å) and angles (°) in SeCN^{t}Bu. Symmetry operation: ^{1} x,y,1/2–z.

Se1	C1		1.741(5)	C11	C13		1.526(4)
N1	C1		1.162(6)	C11	C13^{1}		1.526(4)
N1	C11		1.450(6)	C11	C12		1.521(7)
C1	N1	C11	175.9(5)	N1	C11	C12	106.9(4)
N1	C1	Se1	180.0(5)	C13	C11	C13^{1}	110.5(4)
N1	C11	C13^{1}	107.8(2)	C12	C11	C13	111.8(3)
N1	C11	C13	107.8(2)	C12	C11	C13^{1}	111.8(3)

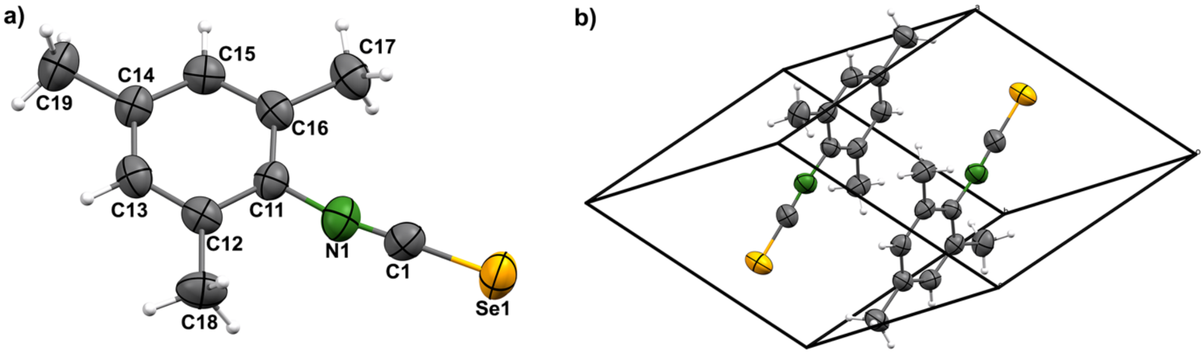

Figure S2. a) Ellipsoid representation of SeCNMesityl with atomic labelling scheme. The thermal ellipsoids are set at a 50% probability level. b) Unit cell plot.

Table S3. Bond lengths (Å) and angles (°) in SeCNMesityl.

Se1	C1		1.720(5)	C11	C16		1.382(6)
N1	C11		1.399(6)	C13	C14		1.386(7)
N1	C1		1.176(6)	C16	C15		1.394(7)
C12	C11		1.402(6)	C16	C17		1.505(6)
C12	C13		1.386(7)	C15	C14		1.375(6)
C12	C18		1.496(7)	C14	C19		1.508(6)
C1	N1	C11	168.8(5)	C11	C16	C15	116.3(4)
C11	C12	C18	121.2(5)	C11	C16	C17	121.7(5)
C13	C12	C11	116.6(5)	C15	C16	C17	121.9(4)
C13	C12	C18	122.2(5)	C14	C15	C16	123.2(4)
N1	C11	C12	118.2(4)	N1	C1	Se1	177.9(5)
C16	C11	N1	118.3(4)	C13	C14	C19	120.6(5)
C16	C11	C12	123.5(4)	C15	C14	C13	117.8(5)
C12	C13	C14	122.6(5)	C15	C14	C19	121.7(5)

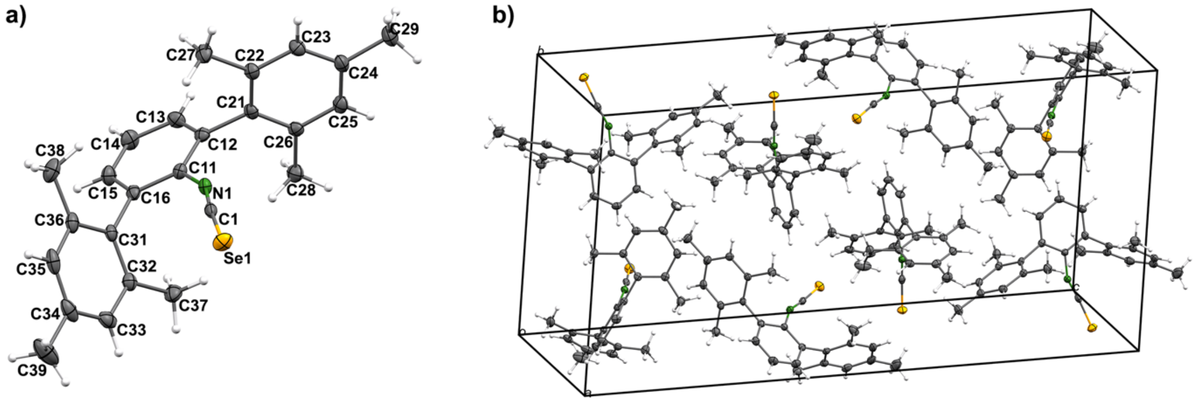

Figure S3. a) Ellipsoid representation of SeCNAr^{Mes2} with atomic labelling scheme. The thermal ellipsoids are set at a 50% probability level. b) Unit cell plot.

Table S4. Bond lengths (Å) and angles (°) in SeCNAr^{Mes2}.

Se1	C1	1.726(3)	C22	C23	1.391(4)
N1	C11	1.387(3)	C22	C27	1.505(4)
N1	C1	1.184(3)	C36	C38	1.514(4)
C11	C12	1.399(4)	C36	C35	1.389(4)
C11	C16	1.400(4)	C13	C14	1.385(4)
C21	C12	1.491(4)	C23	C24	1.389(4)
C21	C26	1.398(4)	C15	C14	1.378(4)
C21	C22	1.407(4)	C32	C33	1.384(4)
C12	C13	1.393(4)	C32	C37	1.510(4)
C16	C31	1.491(4)	C25	C24	1.384(4)
C16	C15	1.393(4)	C35	C34	1.385(4)
C31	C36	1.397(4)	C24	C29	1.508(4)
C31	C32	1.404(4)	C33	C34	1.383(5)
C26	C25	1.393(4)	C34	C39	1.508(5)
C26	C28	1.511(4)			

C1	N1	C11	154.9(2)	C23	C22	C27	121.2(2)
N1	C11	C12	118.2(2)	C31	C36	C38	121.1(2)
N1	C11	C16	119.2(2)	C35	C36	C31	119.0(3)
C12	C11	C16	122.6(2)	C35	C36	C38	120.0(3)
C26	C21	C12	119.3(2)	N1	C1	Se1	175.5(2)
C26	C21	C22	120.4(2)	C14	C13	C12	121.0(2)
C22	C21	C12	120.3(2)	C24	C23	C22	121.9(2)
C11	C12	C21	121.4(2)	C14	C15	C16	121.4(3)
C13	C12	C11	117.5(2)	C31	C32	C37	121.0(3)
C13	C12	C21	121.1(2)	C33	C32	C31	118.6(3)
C11	C16	C31	121.6(2)	C33	C32	C37	120.4(3)
C15	C16	C11	117.3(2)	C24	C25	C26	121.6(3)
C15	C16	C31	121.1(2)	C34	C35	C36	121.9(3)
C36	C31	C16	119.1(2)	C15	C14	C13	120.1(3)
C36	C31	C32	120.1(3)	C23	C24	C29	121.2(3)
C32	C31	C16	120.8(2)	C25	C24	C23	118.5(3)
C21	C26	C28	120.4(2)	C25	C24	C29	120.3(3)
C25	C26	C21	119.0(2)	C34	C33	C32	122.4(3)
C25	C26	C28	120.6(2)	C35	C34	C39	121.2(3)
C21	C22	C27	120.3(2)	C33	C34	C35	117.9(3)
C23	C22	C21	118.5(2)	C33	C34	C39	120.9(3)

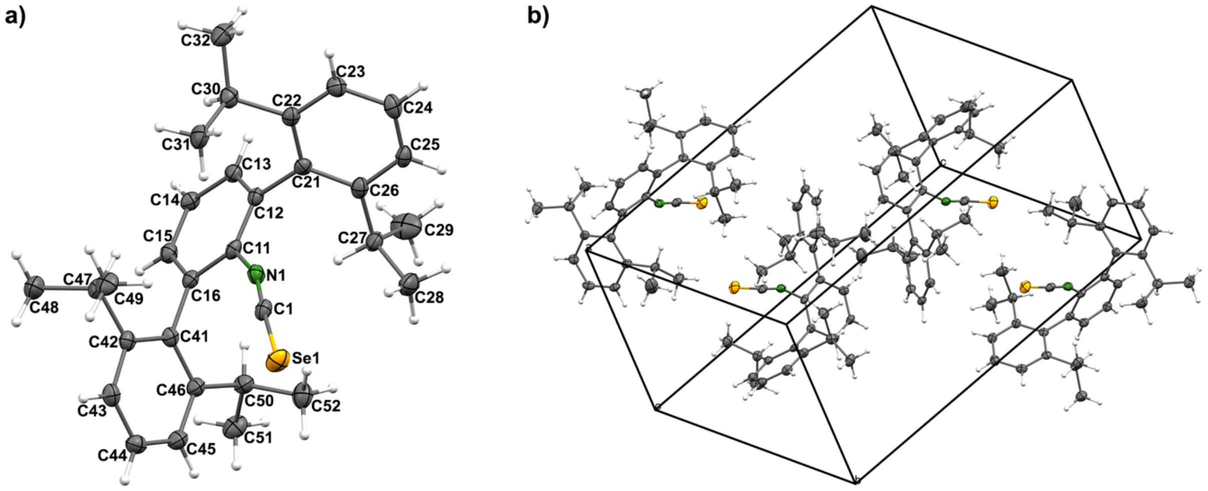

Figure S4. a) Ellipsoid representation of SeCNAr^{Dipp2} with atomic labelling scheme. The thermal ellipsoids are set at a 50% probability level. b) Unit cell plot.

Table S5. Bond lengths (Å) and angles (°) in SeCNAr^{Dipp2}.

Se1	C1	1.744(4)	C21	C26	1.411(5)
N1	C11	1.402(5)	C44	C45	1.375(6)
N1	C1	1.158(5)	C41	C16	1.492(5)
C22	C21	1.412(5)	C41	C42	1.406(5)
C22	C23	1.384(5)	C50	C51	1.530(6)
C22	C30	1.526(5)	C50	C52	1.539(6)
C12	C11	1.391(5)	C42	C47	1.517(5)
C12	C21	1.500(5)	C26	C25	1.394(6)
C12	C13	1.398(5)	C26	C27	1.512(6)
C14	C15	1.374(5)	C24	C25	1.373(6)
C14	C13	1.386(5)	C24	C23	1.397(6)
C46	C45	1.385(5)	C30	C32	1.533(6)
C46	C41	1.411(5)	C30	C31	1.541(6)
C46	C50	1.523(6)	C47	C49	1.531(6)
C15	C16	1.405(5)	C47	C48	1.525(6)
C11	C16	1.399(5)	C27	C28	1.538(6)
C43	C44	1.380(6)	C27	C29	1.514(6)
C43	C42	1.390(6)			

C1	N1	C11	156.7(4)	C46	C50	C52	110.2(3)
C21	C22	C30	120.4(3)	C51	C50	C52	109.6(4)
C23	C22	C21	118.7(4)	C15	C16	C41	122.2(3)
C23	C22	C30	120.8(4)	C11	C16	C15	116.6(4)
C11	C12	C21	122.4(3)	C11	C16	C41	121.2(3)
C11	C12	C13	117.5(3)	C43	C42	C41	118.6(4)
C13	C12	C21	120.0(3)	C43	C42	C47	119.1(4)
C15	C14	C13	121.3(4)	C41	C42	C47	122.3(4)
C45	C46	C41	118.1(4)	C14	C13	C12	120.1(4)
C45	C46	C50	120.1(4)	N1	C1	Se1	176.4(4)
C41	C46	C50	121.7(3)	C21	C26	C27	122.7(4)
C14	C15	C16	120.8(4)	C25	C26	C21	118.1(4)
C12	C11	N1	118.1(3)	C25	C26	C27	119.2(4)
C12	C11	C16	123.7(3)	C25	C24	C23	120.1(4)
C16	C11	N1	118.3(3)	C24	C25	C26	121.5(4)
C44	C43	C42	121.3(4)	C22	C23	C24	120.7(4)

C22	C21	C12	120.3(3)	C22	C30	C32	114.5(3)
C26	C21	C22	120.9(4)	C22	C30	C31	110.2(3)
C26	C21	C12	118.8(3)	C32	C30	C31	108.8(4)
C45	C44	C43	119.4(4)	C42	C47	C49	111.7(3)
C44	C45	C46	122.1(4)	C42	C47	C48	110.9(3)
C46	C41	C16	119.6(3)	C48	C47	C49	111.1(3)
C42	C41	C46	120.5(4)	C26	C27	C28	111.5(4)
C42	C41	C16	119.9(3)	C26	C27	C29	111.1(4)
C46	C50	C51	112.5(4)	C29	C27	C28	111.1(4)

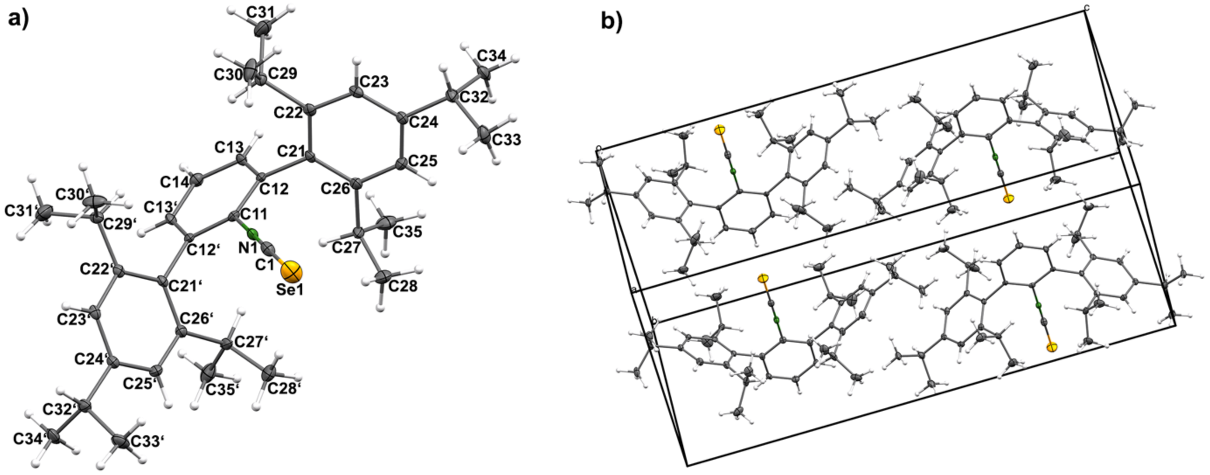

Figure S5. a) Ellipsoid representation of SeCNAr^{Tripp2} with atomic labelling scheme. The thermal ellipsoids are set at a 50% probability level. Symmetry operation: ‘ x,y,0.5–z. b) Unit cell plot.

Table S6. Bond lengths (Å) and angles (°) in SeCNAr^{Tripp2}. Symmetry operation: ‘ x,y,0.5–z.

Se1	C1	1.741(4)	C21	C26	1.399(4)
N1	C11	1.398(5)	C22	C23	1.398(4)
N1	C1	1.154(5)	C22	C29	1.520(4)
C24	C23	1.383(4)	C25	C26	1.395(4)
C24	C25	1.386(4)	C29	C30	1.517(4)
C24	C32	1.522(4)	C29	C31	1.520(4)
C14	C13	1.383(3)	C26	C27	1.522(4)
C14	C13^{1}	1.383(3)	C32	C34	1.520(4)
C12	C13	1.395(4)	C32	C33	1.531(4)
C12	C21	1.499(4)	C27	C28	1.524(4)
C12	C11	1.392(3)	C27	C35	1.531(4)
C21	C22	1.409(4)			

C1	N1	C11	179.7(5)	C12^{1}	C11	N1	118.29(18)
C23	C24	C25	118.2(2)	C12^{1}	C11	C12	123.4(4)
C23	C24	C32	120.2(3)	C24	C25	C26	122.0(3)
C25	C24	C32	121.6(3)	C22	C29	C31	111.2(2)
C13	C14	C13^{1}	120.8(4)	C30	C29	C22	111.3(2)
C13	C12	C21	121.2(2)	C30	C29	C31	111.3(3)
C11	C12	C13	117.4(3)	N1	C1	Se1	179.4(4)

C11	C12	C21	121.4(3)	C21	C26	C27	122.0(2)
C14	C13	C12	120.5(3)	C25	C26	C21	118.6(3)
C22	C21	C12	119.8(2)	C25	C26	C27	119.3(3)
C26	C21	C12	119.5(2)	C24	C32	C33	112.7(2)
C26	C21	C22	120.7(2)	C34	C32	C24	110.5(2)
C21	C22	C29	122.4(2)	C34	C32	C33	110.4(3)
C23	C22	C21	118.0(3)	C26	C27	C28	112.5(3)
C23	C22	C29	119.6(3)	C26	C27	C35	110.6(2)
C24	C23	C22	122.4(3)	C28	C27	C35	111.0(3)
C12	C11	N1	118.29(18)				

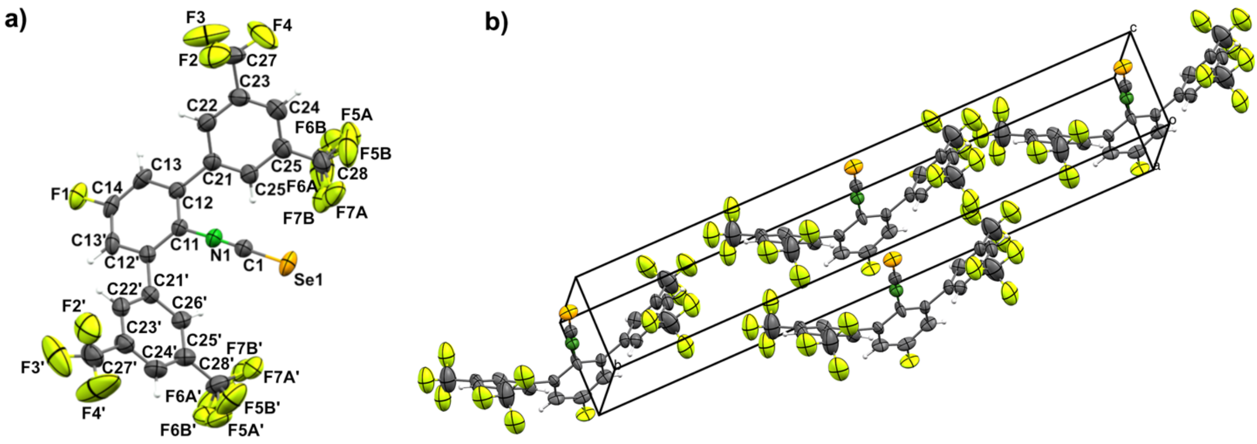

Figure S6. a) Ellipsoid representation of SeCN*p*-FAr^{DarF2} with atomic labelling scheme. The thermal ellipsoids are set at a 50% probability level. Symmetry operation: ' x,–y,z. b) Unit cell plot.

Table S7. Bond lengths (Å) and angles (°) in SeCN*p*-FAr^{DarF2}. Symmetry operation: ' x,–y,z.

Se1	C1		1.716(12)	C24	C23		1.379(15)
F1	C14		1.345(19)	C23	C22		1.374(14)
N1	C1		1.143(17)	C23	C27		1.513(13)
N1	C11		1.398(19)	C27	F2		1.331(18)
C11	C12		1.396(11)	C27	F4		1.23(2)
C11	C12[1]		1.396(11)	C27	F3		1.28(2)
C12	C13		1.392(9)	C28	F5A		1.33(2)
C12	C21		1.506(12)	C28	F6A		1.29(3)
C13	C14		1.384(13)	C28	F7A		1.32(2)
C21	C26		1.373(12)	C28	F5B		1.41(5)
C21	C22		1.409(13)	C28	F7B		1.36(4)
C26	C25		1.398(13)	C28	F6B		1.22(3)
C25	C24		1.385(13)				
C25	C28		1.49(2)				
C1	N1	C11	172.3(14)	C22	C23	C24	121.5(9)
N1	C1	Se1	179.8(12)	C22	C23	C27	117.6(10)
C12	C11	N1	119.6(7)	C23	C22	C21	119.4(8)
C12[1]	C11	N1	119.6(7)	F2	C27	C23	111.5(11)
C12[1]	C11	C12	120.8(15)	F4	C27	C23	113.8(14)

C11	C12	C21	120.8(10)	F4	C27	F2	107.8(19)
C13	C12	C11	120.1(11)	F4	C27	F3	107.8(17)
C13	C12	C21	119.1(9)	F3	C27	C23	114.4(15)
C14	C13	C12	117.1(11)	F3	C27	F2	100.6(15)
F1	C14	C13^{1}	117.5(8)	F5A	C28	C25	113.0(15)
F1	C14	C13	117.5(8)	F6A	C28	C25	114.7(18)
C13	C14	C13^{1}	124.9(15)	F6A	C28	F5A	104(2)
C26	C21	C12	120.7(8)	F6A	C28	F7A	108(2)
C26	C21	C22	119.0(8)	F7A	C28	C25	111.0(12)
C22	C21	C12	120.3(8)	F7A	C28	F5A	105(2)
C21	C26	C25	121.1(9)	F5B	C28	C25	103(3)
C26	C25	C28	119.1(10)	F7B	C28	C25	115.5(15)
C24	C25	C26	119.5(10)	F7B	C28	F5B	123(4)
C24	C25	C28	121.1(9)	F6B	C28	C25	113(2)
C23	C24	C25	119.4(9)	F6B	C28	F5B	99(4)
C24	C23	C27	120.8(10)	F6B	C28	F7B	104(3)

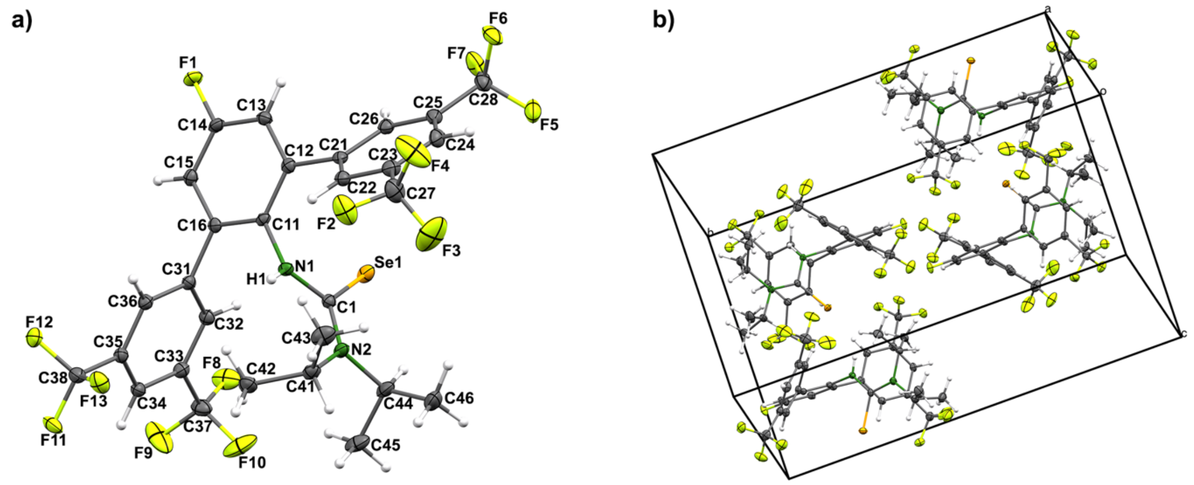

Figure S7. a) Ellipsoid representation of (i-Prop)$_2$NC(Se)NHp-FAr^{DarF2} with atomic labelling scheme. The thermal ellipsoids are set at a 50% probability level. b) Unit cell plot.

Table S8. Bond lengths (Å) and angles (°) in (i-Prop)$_2$NC(Se)NH**p**-FAr^{DarF2}.

Se1	C1	1.8588(17)	C14	C15	1.374(2)
F12	C38	1.346(2)	C36	C31	1.390(2)
F1	C14	1.3581(19)	C36	C35	1.389(2)
F11	C38	1.340(2)	C21	C26	1.394(2)
F13	C38	1.340(2)	C21	C22	1.393(2)
F5	C28	1.343(2)	C15	C16	1.392(2)
F6	C28	1.344(2)	C16	C31	1.495(2)
F7	C28	1.336(2)	C32	C31	1.393(2)
F8	C37	1.325(2)	C32	C33	1.387(2)
F2	C27	1.329(2)	C34	C33	1.389(2)
N1	C1	1.354(2)	C34	C35	1.386(2)
N1	C11	1.432(2)	C26	C25	1.390(2)
F10	C37	1.339(2)	C33	C37	1.501(2)
F9	C37	1.328(2)	C35	C38	1.497(2)

N40	C1	1.337(2)		C22	C23	1.393(2)
N40	C41	1.491(2)		C41	C42	1.529(3)
N40	C44	1.486(2)		C41	C43	1.526(3)
F4	C27	1.330(2)		C23	C24	1.382(3)
F3	C27	1.331(3)		C23	C27	1.497(3)
C12	C11	1.400(2)		C24	C25	1.388(3)
C12	C13	1.394(2)		C25	C28	1.499(3)
C12	C21	1.489(2)		C44	C45	1.523(3)
C11	C16	1.398(2)		C44	C46	1.525(3)
C13	C14	1.377(2)				

C1	N1	C11	124.13(14)	N40	C41	C42	114.16(15)
C1	N40	C41	124.47(14)	N40	C41	C43	112.95(15)
C1	N40	C44	120.62(14)	C43	C41	C42	113.47(16)
C44	N40	C41	114.83(13)	C22	C23	C27	120.45(17)
N1	C1	Se1	117.20(12)	C24	C23	C22	120.81(17)
N40	C1	Se1	124.85(12)	C24	C23	C27	118.74(16)
N40	C1	N1	117.95(15)	F12	C38	C35	111.97(14)
C11	C12	C21	122.48(14)	F11	C38	F12	106.40(14)
C13	C12	C11	118.65(15)	F11	C38	C35	113.28(14)
C13	C12	C21	118.85(15)	F13	C38	F12	106.25(14)
C12	C11	N1	120.76(14)	F13	C38	F11	106.59(14)
C16	C11	N1	118.27(14)	F13	C38	C35	111.88(14)
C16	C11	C12	120.95(15)	C23	C24	C25	119.33(16)
C14	C13	C12	119.09(15)	C26	C25	C28	120.77(16)
F1	C14	C13	118.10(15)	C24	C25	C26	120.45(17)
F1	C14	C15	118.56(15)	C24	C25	C28	118.77(16)
C15	C14	C13	123.34(15)	F5	C28	F6	105.68(14)
C35	C36	C31	120.44(16)	F5	C28	C25	112.24(16)
C26	C21	C12	119.00(15)	F6	C28	C25	111.76(15)
C22	C21	C12	121.63(15)	F7	C28	F5	106.45(15)
C22	C21	C26	119.34(15)	F7	C28	F6	107.07(15)
C14	C15	C16	118.07(15)	F7	C28	C25	113.16(15)
C11	C16	C31	120.55(14)	F8	C37	F10	106.11(16)
C15	C16	C11	119.87(15)	F8	C37	F9	106.86(16)
C15	C16	C31	119.58(15)	F8	C37	C33	113.32(15)
C33	C32	C31	120.13(16)	F10	C37	C33	111.65(16)
C36	C31	C16	119.97(15)	F9	C37	F10	105.98(17)
C36	C31	C32	118.95(15)	F9	C37	C33	112.41(16)
C32	C31	C16	121.08(15)	N40	C44	C45	110.11(15)
C35	C34	C33	118.60(16)	N40	C44	C46	111.44(16)
C25	C26	C21	120.21(16)	C45	C44	C46	113.59(16)
C32	C33	C34	121.05(16)	F2	C27	F4	106.87(18)
C32	C33	C37	119.99(16)	F2	C27	F3	106.13(18)
C34	C33	C37	118.93(16)	F2	C27	C23	113.46(16)
C36	C35	C38	117.79(15)	F4	C27	F3	105.77(17)
C34	C35	C36	120.81(16)	F4	C27	C23	112.15(17)
C34	C35	C38	121.36(15)	F3	C27	C23	111.94(18)
C21	C22	C23	119.85(16)				

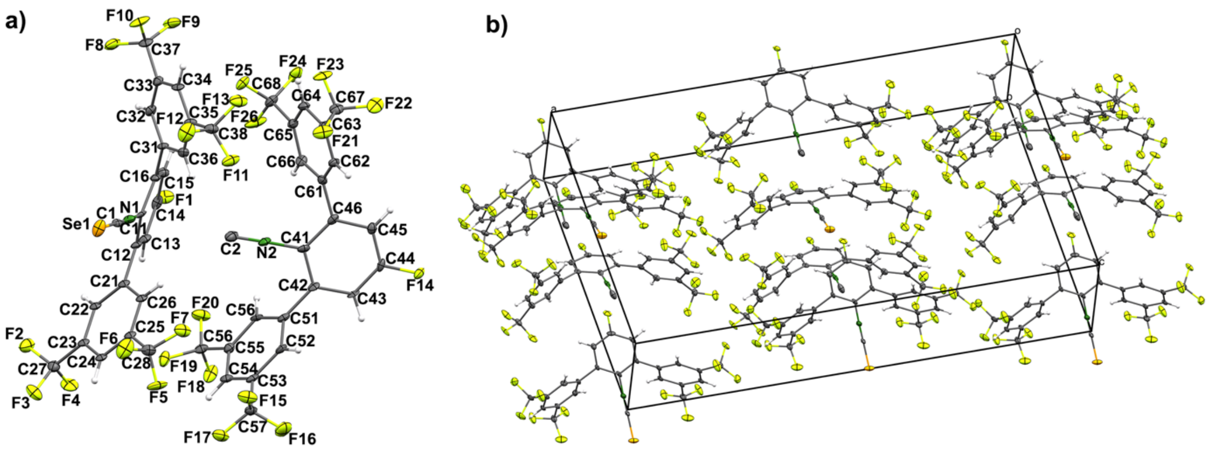

Figure S8. a) Ellipsoid representation of SeCN*p*-FAr^{DarF2} x CN*p*-FAr^{DarF2} with atomic labelling scheme. The thermal ellipsoids are set at a 50% probability level. Symmetry operation: ‘ x,–y,z. b) Unit cell plot.

Table S9. Bond lengths (Å) and angles (°) in SeCN*p*-FAr^{DarF2} x CN*p*-FAr^{DarF2}. Symmetry operation: ‘ x,–y,z.

Se1	C1	1.812(6)	C62	C61	1.400(9)
F13	C38	1.401(8)	C68	C65	1.560(9)
F3	C27	1.322(8)	C51	C52	1.469(8)
F7	C28	1.431(8)	C51	C56	1.461(8)
F5	C28	1.316(8)	C51	C42	1.556(8)
F6	C28	1.362(8)	C52	C53	1.472(9)
F26	C68	1.336(8)	C53	C54	1.444(9)
F25	C68	1.332(7)	C53	C57	1.578(8)
F24	C68	1.430(8)	C54	C55	1.443(9)
F21	C67	1.437(8)	C55	C56	1.473(8)
F23	C67	1.302(7)	C55	C58	1.560(9)
F22	C67	1.393(8)	C14	C13	1.476(9)
F16	C57	1.337(8)	C13	C12	1.430(9)
F17	C57	1.414(7)	C12	C21	1.564(8)
F15	C57	1.345(8)	C31	C36	1.448(9)
F18	C58	1.312(7)	C31	C32	1.392(8)
F19	C58	1.413(8)	C36	C35	1.391(8)
F20	C58	1.386(8)	C35	C34	1.395(9)
F1	C14	1.373(7)	C35	C38	1.551(9)
F14	C44	1.391(7)	C34	C33	1.428(9)
N1	C1	1.167(8)	C33	C32	1.391(9)
N1	C11	1.455(8)	C33	C37	1.510(8)
N2	C2	1.197(10)	C21	C22	1.447(8)
N2	C41	1.417(8)	C21	C26	1.439(8)
F11	C38	1.323(8)	C22	C23	1.480(8)
F12	C38	1.383(9)	C23	C24	1.424(9)
F9	C37	1.424(8)	C23	C27	1.550(9)
F10	C37	1.306(7)	C24	C25	1.450(9)
F8	C37	1.397(8)	C25	C26	1.470(8)
F2	C27	1.362(8)	C25	C28	1.563(9)
F4	C27	1.428(8)	C41	C46	1.502(9)
C16	C11	1.485(8)	C41	C42	1.415(8)

C16	C15	1.432(9)	C46	C45	1.433(9)
C16	C31	1.487(8)	C46	C61	1.486(8)
C11	C12	1.387(8)	C45	C44	1.364(9)
C15	C14	1.381(9)	C44	C43	1.444(9)
C64	C63	1.432(9)	C43	C42	1.442(9)
C64	C65	1.401(9)	C61	C66	1.429(9)
C63	C62	1.358(9)	C66	C65	1.371(9)
C63	C67	1.523(8)			

C1	N1	C11	175.0(6)	C34	C35	C38	120.4(5)
C2	N2	C41	174.1(6)	C35	C34	C33	120.1(5)
C11	C16	C31	122.3(5)	C34	C33	C37	120.8(5)
C15	C16	C11	120.5(5)	C32	C33	C34	121.1(6)
C15	C16	C31	117.1(5)	C32	C33	C37	117.9(6)
N1	C1	Se1	179.7(6)	C33	C32	C31	118.5(6)
N1	C11	C16	121.2(5)	F13	C38	C35	111.4(5)
C12	C11	N1	115.6(5)	F11	C38	F13	104.5(6)
C12	C11	C16	123.1(5)	F11	C38	F12	103.0(5)
C14	C15	C16	116.4(6)	F11	C38	C35	114.4(5)
C65	C64	C63	120.1(5)	F12	C38	F13	112.5(6)
C64	C63	C67	122.7(5)	F12	C38	C35	110.7(5)
C62	C63	C64	120.5(6)	F9	C37	C33	114.7(5)
C62	C63	C67	116.7(6)	F10	C37	F9	106.6(5)
C63	C62	C61	118.5(6)	F10	C37	F8	107.6(5)
F26	C68	F24	109.3(6)	F10	C37	C33	110.4(5)
F26	C68	C65	113.6(5)	F8	C37	F9	106.7(5)
F25	C68	F26	103.8(5)	F8	C37	C33	110.5(5)
F25	C68	F24	108.4(5)	C22	C21	C12	121.8(5)
F25	C68	C65	110.4(5)	C26	C21	C12	119.7(5)
F24	C68	C65	111.1(5)	C26	C21	C22	118.6(5)
F21	C67	C63	108.0(5)	C21	C22	C23	121.8(6)
F23	C67	F21	108.4(5)	C22	C23	C27	120.2(6)
F23	C67	F22	106.2(5)	C24	C23	C22	119.9(5)
F23	C67	C63	110.9(5)	C24	C23	C27	119.8(5)
F22	C67	F21	108.9(5)	C23	C24	C25	117.9(5)
F22	C67	C63	114.3(5)	C24	C25	C26	122.9(5)
C52	C51	C42	121.5(5)	C24	C25	C28	118.2(5)
C56	C51	C52	116.4(5)	C26	C25	C28	118.9(5)
C56	C51	C42	122.1(5)	C21	C26	C25	118.9(5)
C51	C52	C53	122.4(5)	F3	C27	F2	103.6(6)
C52	C53	C57	119.8(5)	F3	C27	F4	107.7(5)
C54	C53	C52	120.8(6)	F3	C27	C23	110.0(5)
C54	C53	C57	119.4(5)	F2	C27	F4	105.4(5)
C55	C54	C53	116.9(6)	F2	C27	C23	114.2(5)
C54	C55	C56	123.4(6)	F4	C27	C23	115.0(5)
C54	C55	C58	116.5(5)	F7	C28	C25	111.0(5)
C56	C55	C58	120.1(5)	F5	C28	F7	107.3(6)
C51	C56	C55	120.0(5)	F5	C28	F6	103.8(6)
F16	C57	F17	110.5(5)	F5	C28	C25	112.2(5)
F16	C57	F15	104.3(5)	F6	C28	F7	109.7(5)
F16	C57	C53	110.1(5)	F6	C28	C25	112.4(5)
F17	C57	C53	114.6(5)	N2	C41	C46	124.3(5)

F15	C57	F17	105.0(5)	C42	C41	N2	115.4(5)
F15	C57	C53	111.7(5)	C42	C41	C46	120.0(5)
F18	C58	F19	104.9(5)	C45	C46	C41	122.6(5)
F18	C58	F20	104.8(5)	C45	C46	C61	117.0(5)
F18	C58	C55	111.1(5)	C61	C46	C41	120.3(5)
F19	C58	C55	113.2(5)	C44	C45	C46	116.4(5)
F20	C58	F19	111.0(5)	F14	C44	C43	122.8(5)
F20	C58	C55	111.4(5)	C45	C44	F14	115.3(5)
F1	C14	C15	116.1(6)	C45	C44	C43	121.9(5)
F1	C14	C13	121.1(6)	C42	C43	C44	124.5(5)
C15	C14	C13	122.7(6)	C41	C42	C51	121.2(5)
C12	C13	C14	121.6(6)	C41	C42	C43	114.6(5)
C11	C12	C13	115.5(5)	C43	C42	C51	124.1(5)
C11	C12	C21	121.6(5)	C62	C61	C46	117.4(5)
C13	C12	C21	122.8(5)	C62	C61	C66	122.3(6)
C36	C31	C16	119.9(5)	C66	C61	C46	120.3(5)
C32	C31	C16	118.9(5)	C65	C66	C61	118.2(6)
C32	C31	C36	121.2(5)	C64	C65	C68	119.6(5)
C35	C36	C31	119.2(5)	C66	C65	C64	120.3(6)
C36	C35	C34	119.9(6)	C66	C65	C68	120.1(6)
C36	C35	C38	119.7(5)				

Selected spectroscopic data

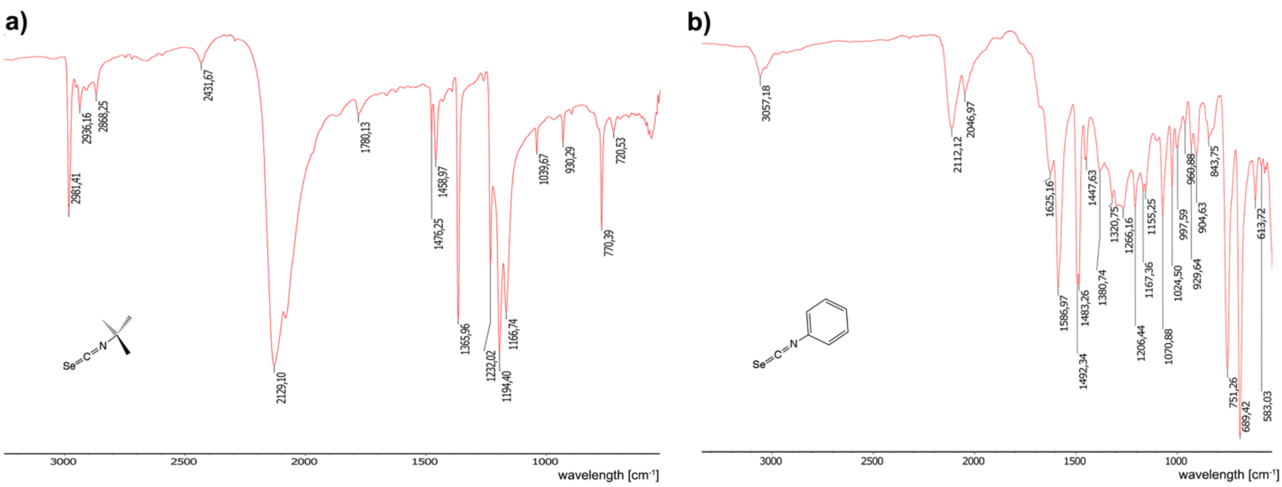

Figure S9. IR spectra (ATR) of a) SeCN^{t}Bu and b) SeCNPh.

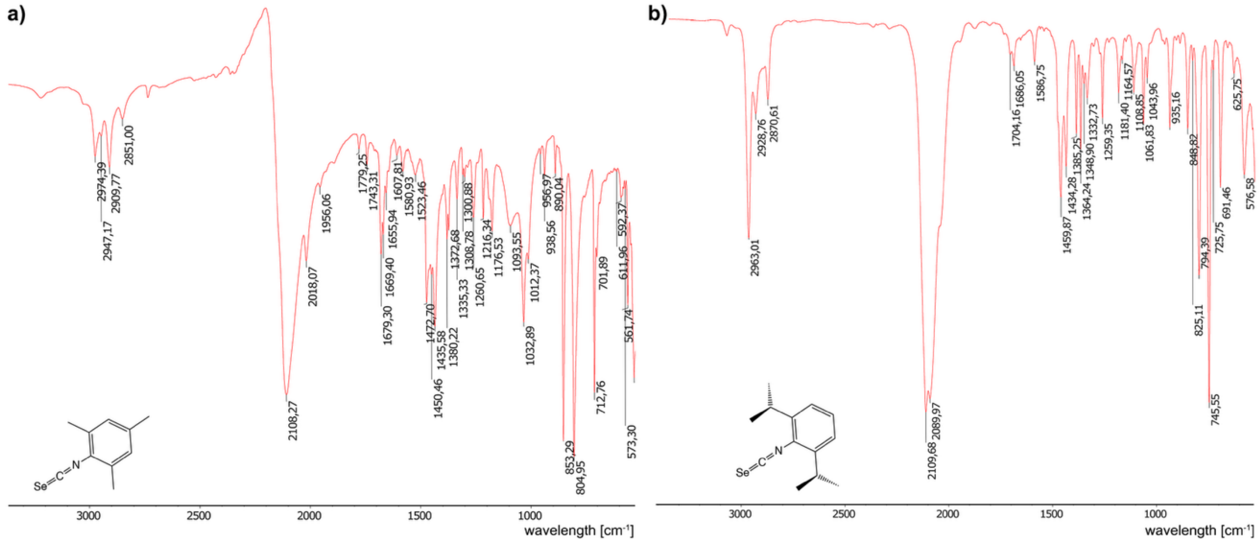

Figure S10. IR spectra (ATR) of a) SeCNMesityl and b) SeCN$^{i\text{-}prop2}$Ph.

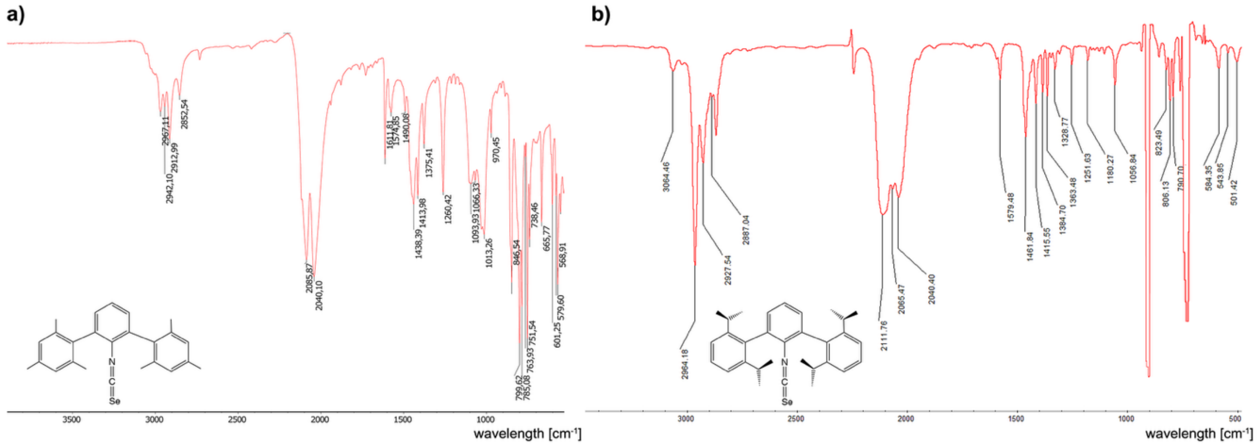

Figure S11. IR spectra (ATR) of a) SeCNAr^{Mes2} and b) SeCNAr^{Dipp2}.

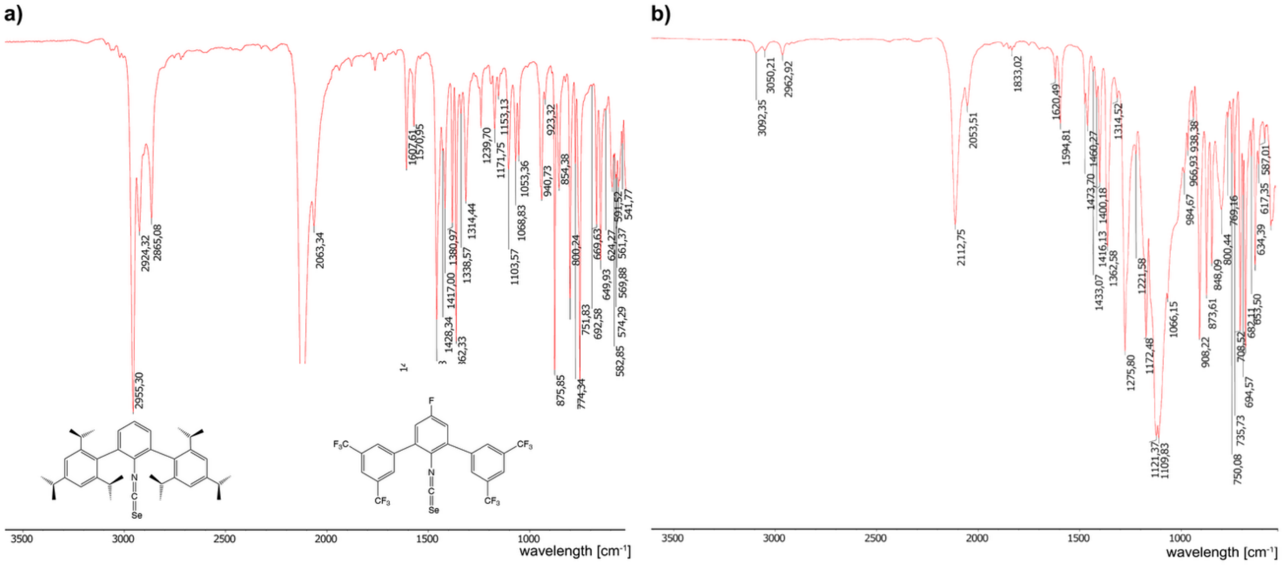

Figure S12. IR spectra (ATR) of a) SeCNAr^{Tripp2} and b) SeCN*p*-FAr^{DarF2}.

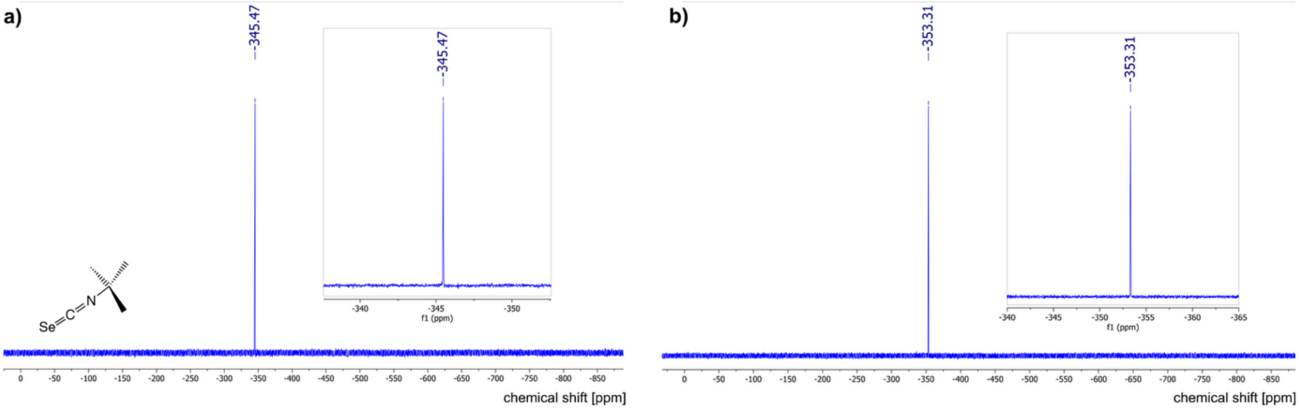

Figure S13. ^{77}Se NMR spectra of SeCN^{t}Bu in $CDCl_3$ (a) and THF-d_8 (b).

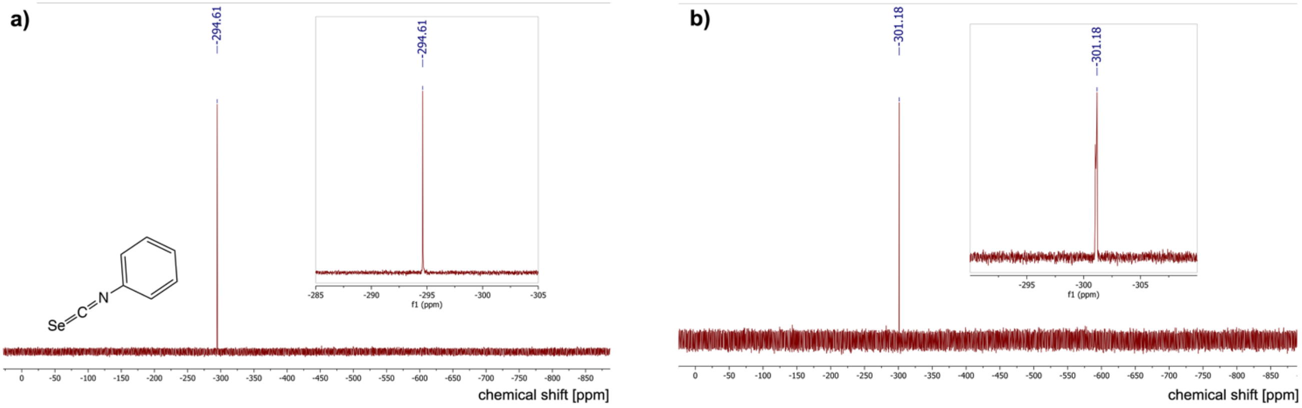

Figure S14. ^{77}Se NMR spectra of SeCNPh in $CDCl_3$ (a) and THF-d_8 (b).

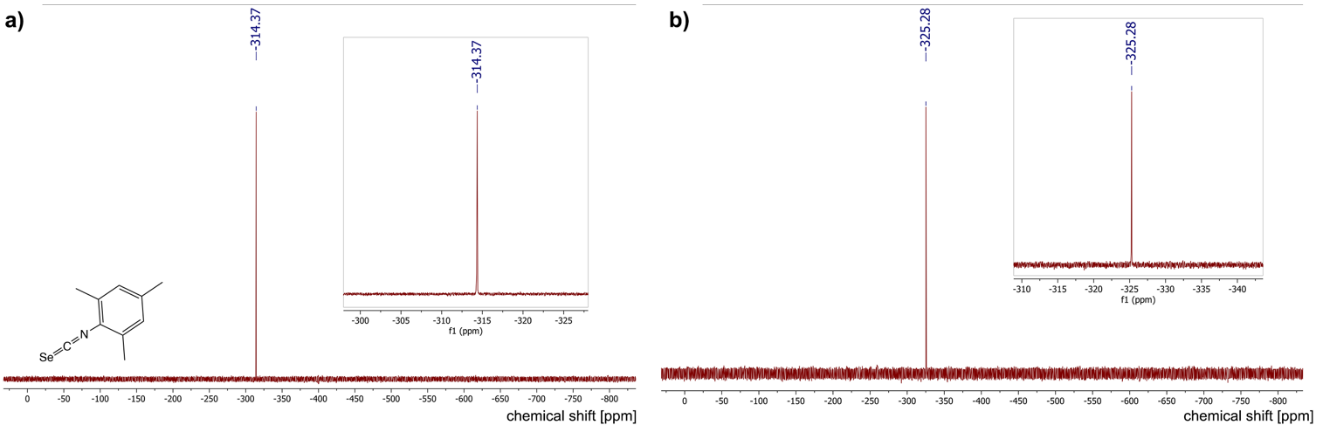

Figure S15. ^{77}Se NMR spectra of SeCNMesityl in $CDCl_3$ (a) and THF-d_8 (b).

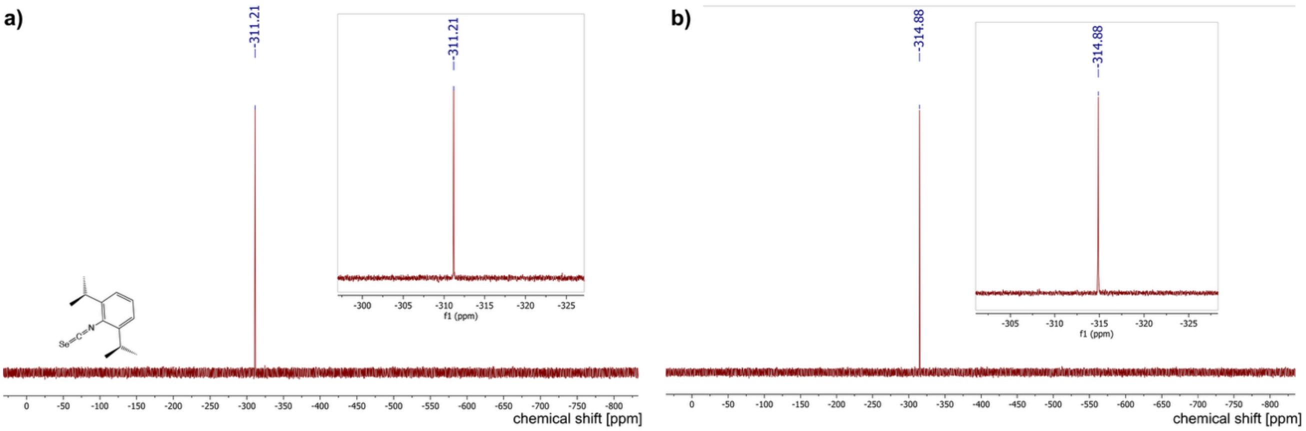

Figure S16. ^{77}Se NMR spectra of SeCN(2,6-diisoprop)phenyl in $CDCl_3$ (a) and THF-d_8 (b).

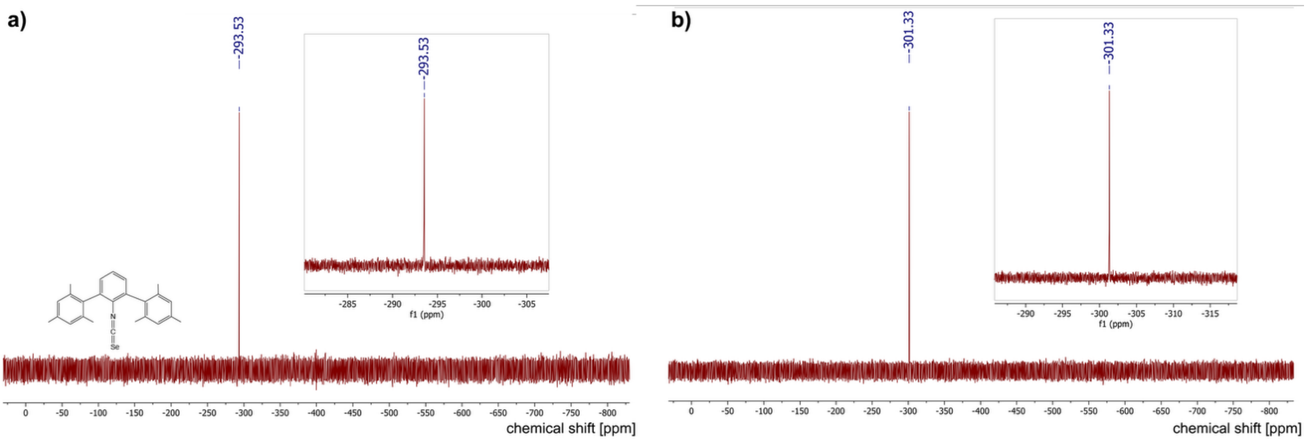

Figure S17. ^{77}Se NMR spectra of SeCNAr^{Mes2} in toluene-d_8 (a) and THF-d_8 (b).

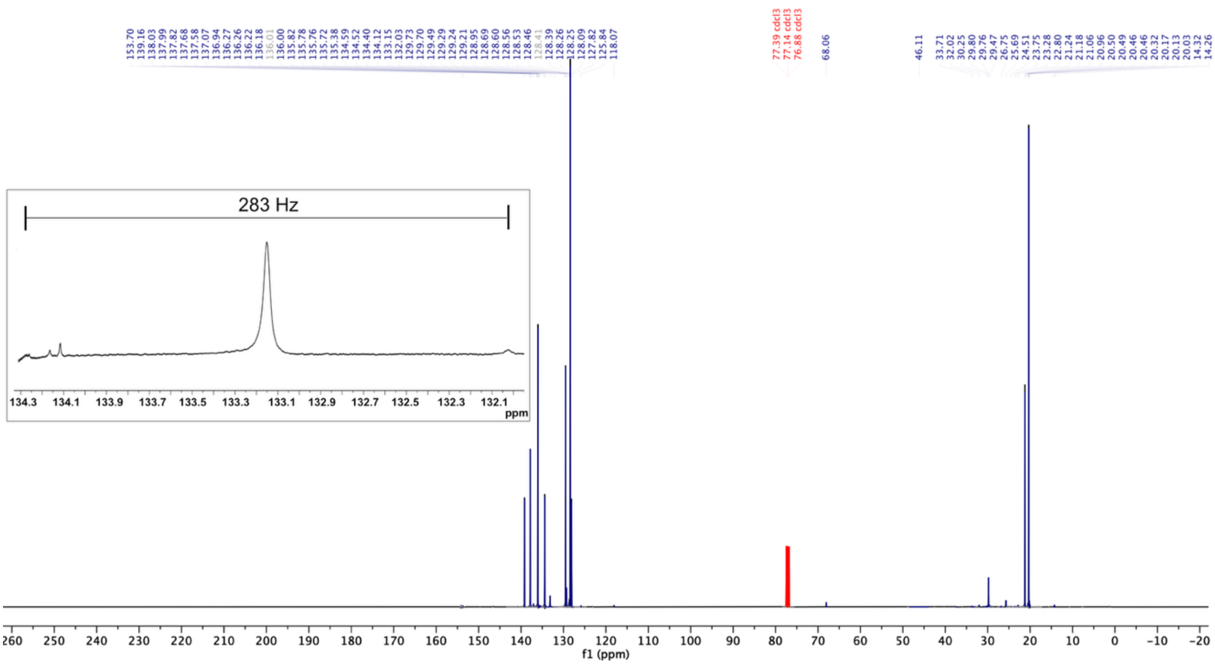

Figure S18. ^{13}C NMR spectrum of SeCNAr^{Mes2} in $CDCl_3$.

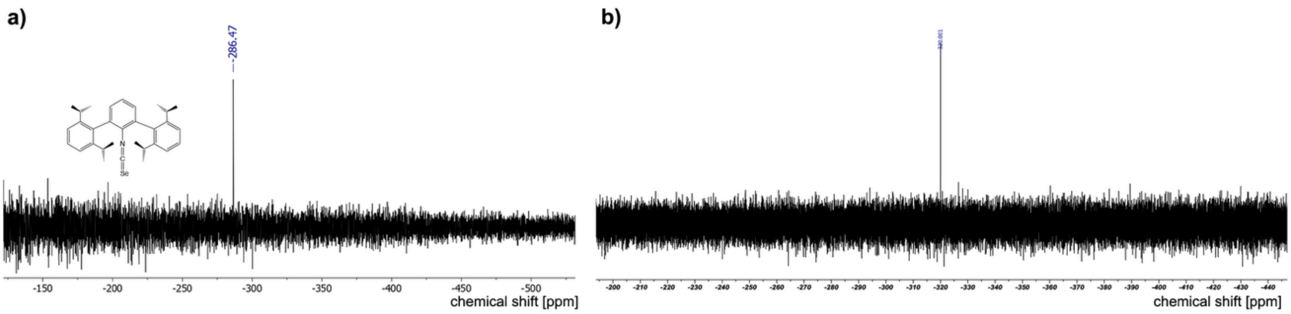

Figure S19. ^{77}Se NMR spectra of SeCNAr^{Dipp2} in $CDCl_3$ (a) and THF-d_8 (b).

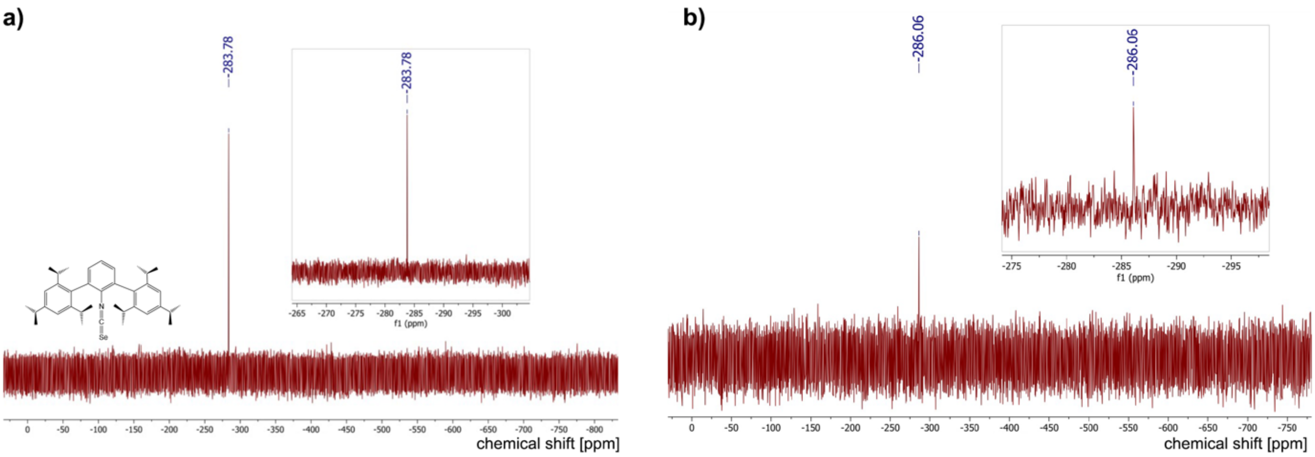

Figure S20. ^{77}Se NMR spectra of SeCNAr^{Tripp2} in $CDCl_3$ (a) THF-d_8 (b).

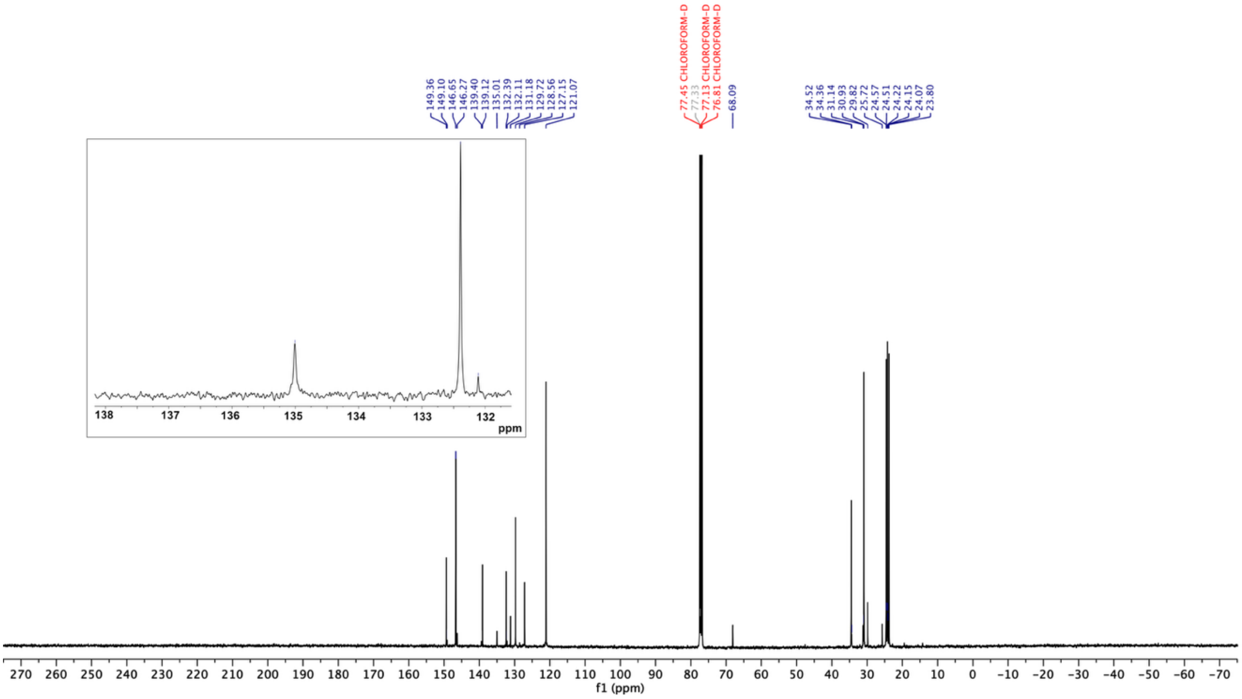

Figure S21. ^{13}C NMR spectrum of SeCNAr^{Tripp2} in $CDCl_3$.

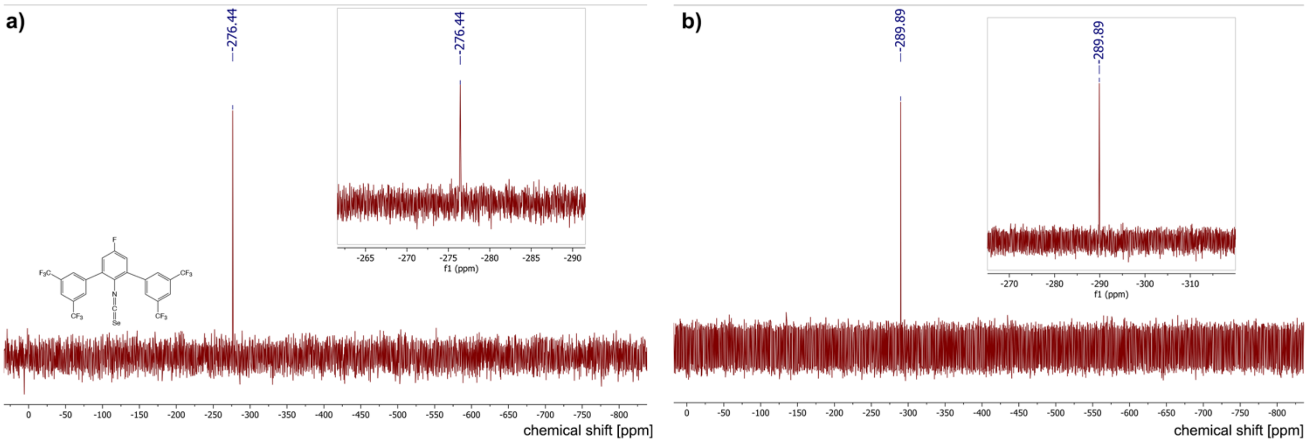

Figure S22. ^{77}Se NMR spectra of SeCN*p*-FAr^{DarF2} in toluene (a) THF-d_8 (b).

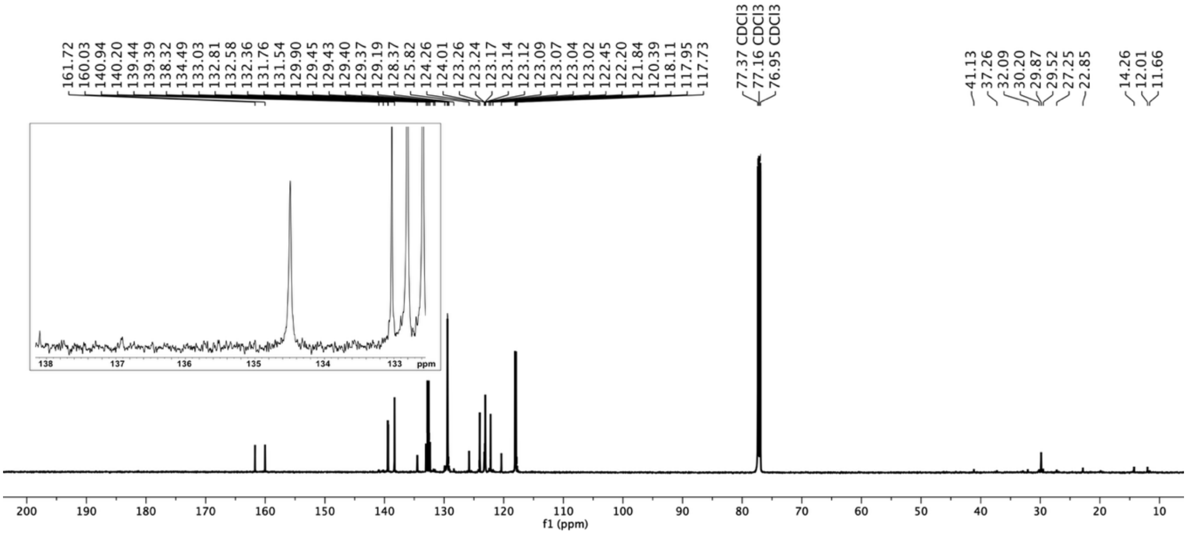

Figure S23. ^{13}C NMR spectrum of SeCN*p*-FAr^{DarF2} in $CDCl_3$.

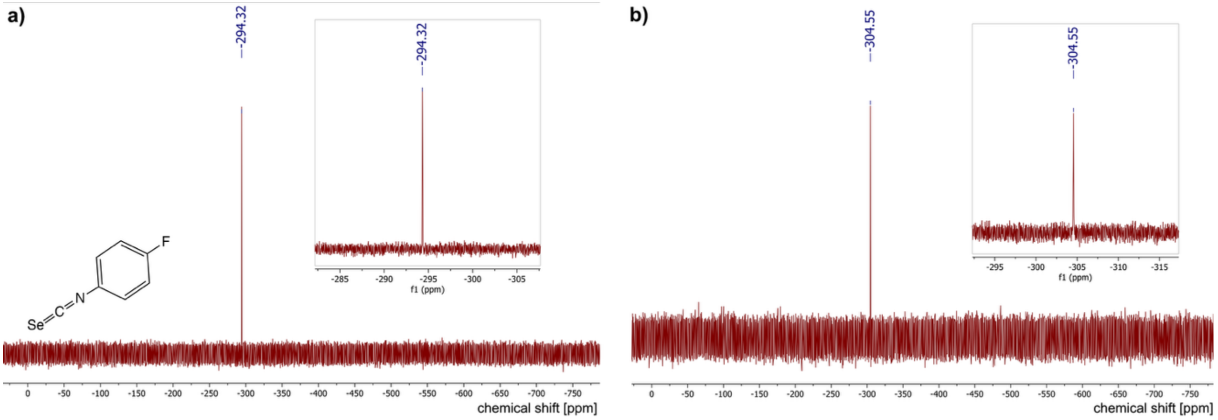

Figure S24. ^{77}Se NMR spectra of SeCNPhF in $CDCl_3$ (a) THF-d_8 (b).

Table S10. ^{77}Se NMR chemical shifts (ppm) and ν_{CN} IR frequencies (cm^{-1}) of the isoselenocyanates and the corresponding isocyanides.

	^{77}Se NMR (ppm)			ν_{CN} (cm^{-1})	
	$CDCl_3$	toluene	THF	SeCNR	CNR
SeCN^{t}Bu	–345.5		–353.3	2129, 2082	2135
SeCNMesityl	–314.4		–325.3	2108, 2018	
SeCN$^{i\text{-Prop2}}$Ph	–311.2		–314.9	2110, 2090	
SeCNAr^{Mes2}	–294.3	–293.5	–301.3	2086, 2040	2112
SeCNPhF	–294.3		–304.6		2129
SeCNPh	–294.6		–301.2	2112, 2047	2180
SeCNAr^{Dipp2}	–288.5		–320.0	2121, 2040	2124
SeCNAr^{Tripp2}	–283.8	–286.1	–319.2	2112, 2063	2112
SeCN*p*-FAr^{DarF2}	–274.1	–276.4	–289.9	2113, 2053	2121

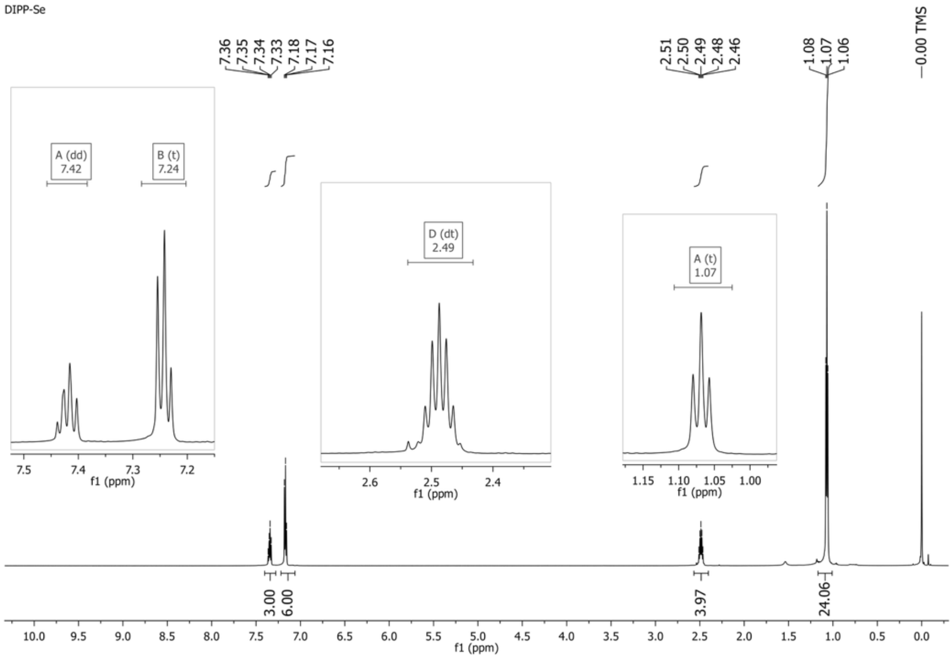

Figure S25. ^{1}H NMR spectrum of ^{13}C-enriched Se*CNAr^{Dipp} in $CDCl_3$.

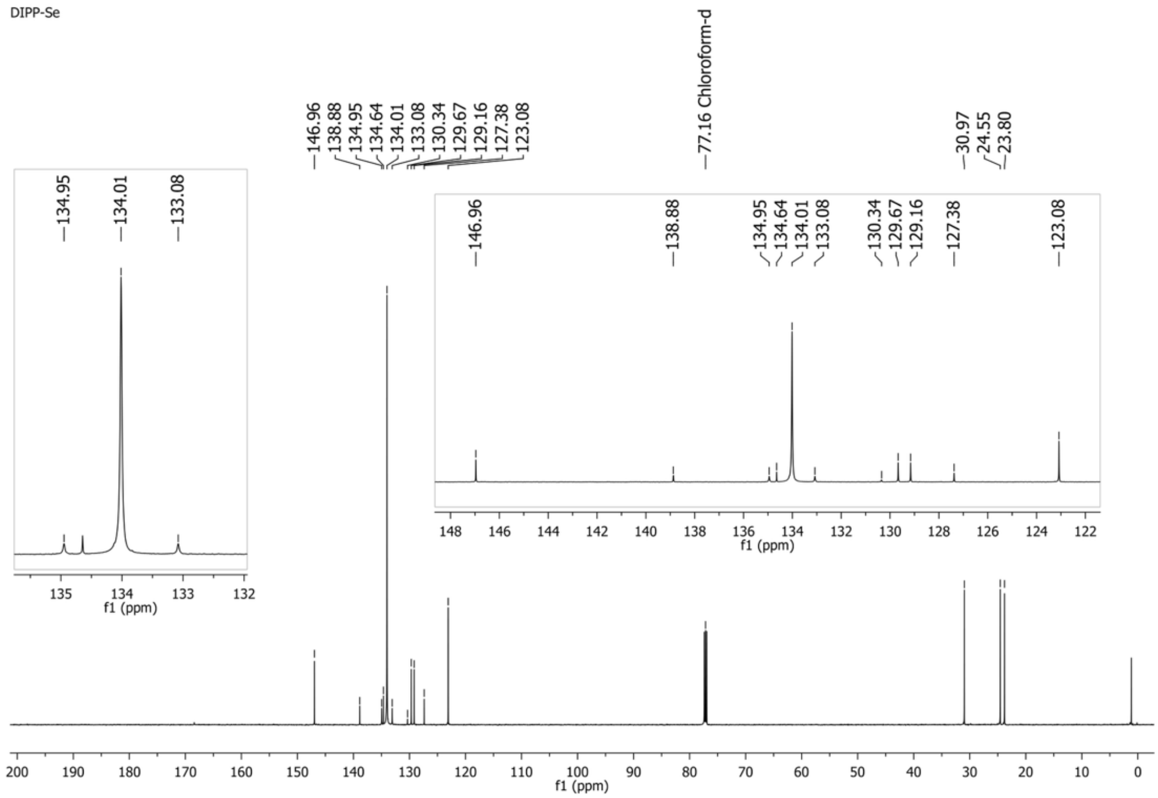

Figure S26. ^{13}C NMR spectrum of ^{13}C-enriched Se*CNAr^{Dipp} in $CDCl_3$.

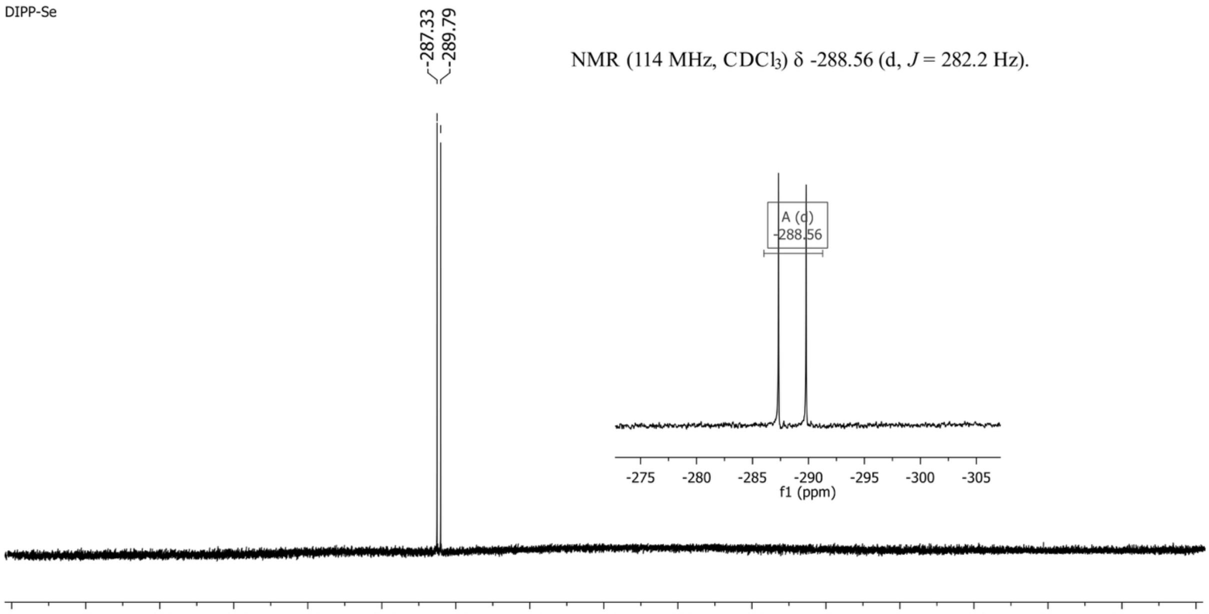

Figure S27. ^{77}Se NMR spectrum of ^{13}C-enriched Se*CNAr^{Dipp} in $CDCl_3$.

Computational data

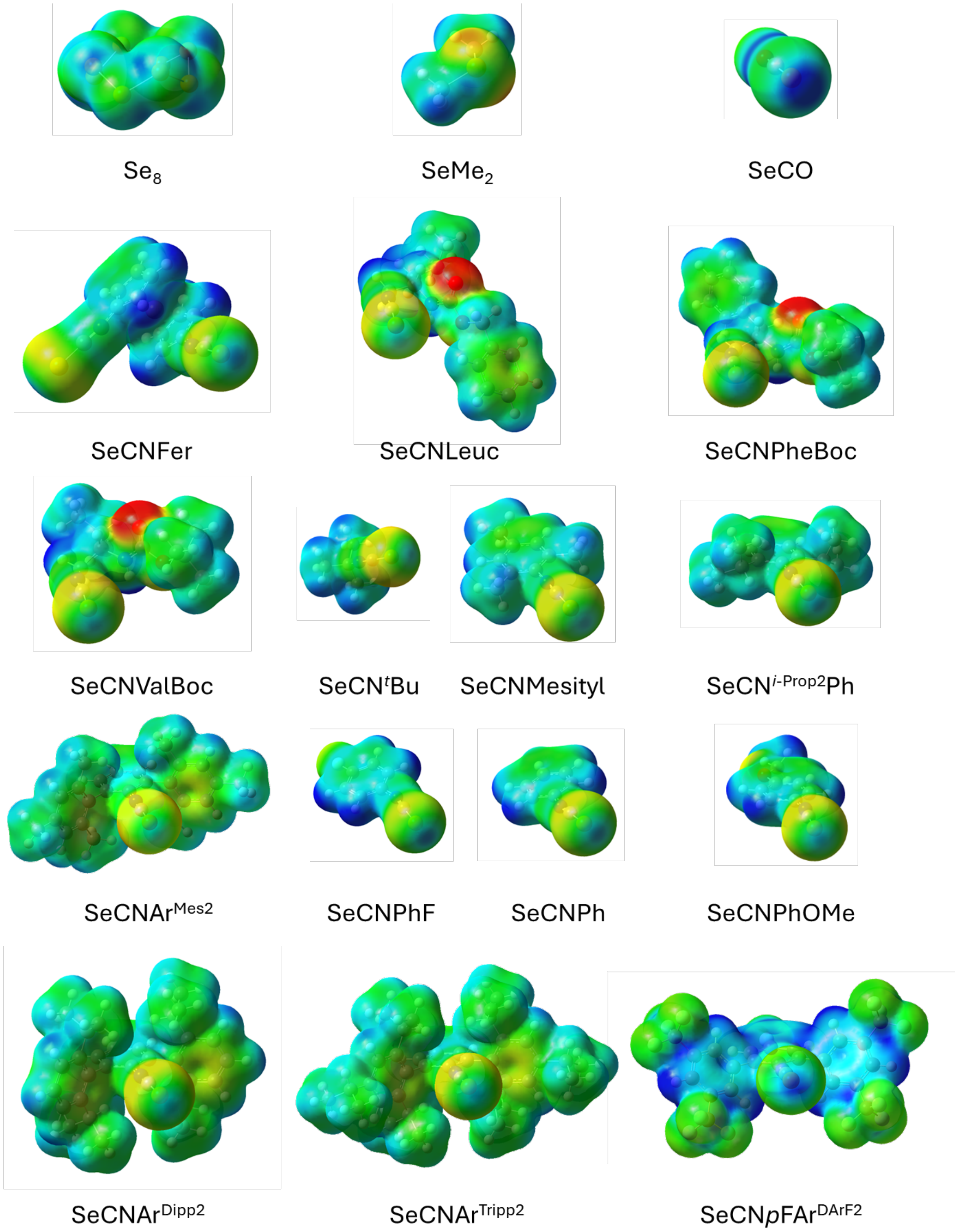

Figure S28. Molecular electrostatic potential mapping of some representative isoselenocyanates, Se_8, $SeMe_2$ and SeCO (depicted color scaled to the boundary surface potential maxima of SeCN^{t}Bu) at an isosurface electron density level of 0.004 e/A^3.

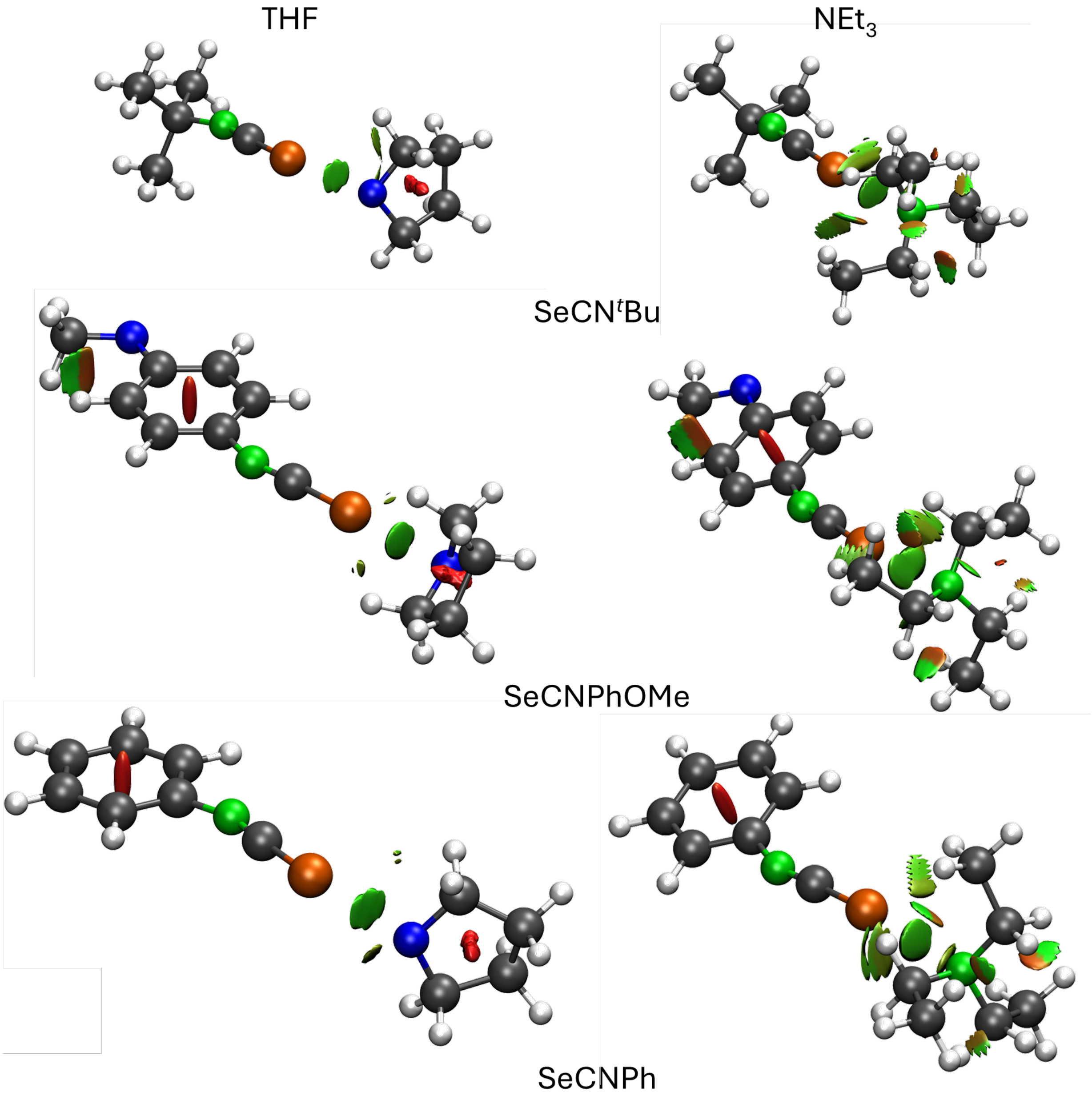

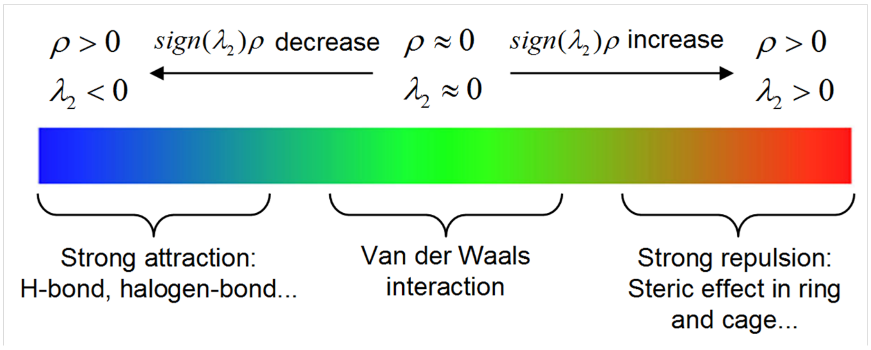

Figure S29. Reduced-density gradient (RDG) analysis for the interaction of isoselenocyanates with THF.

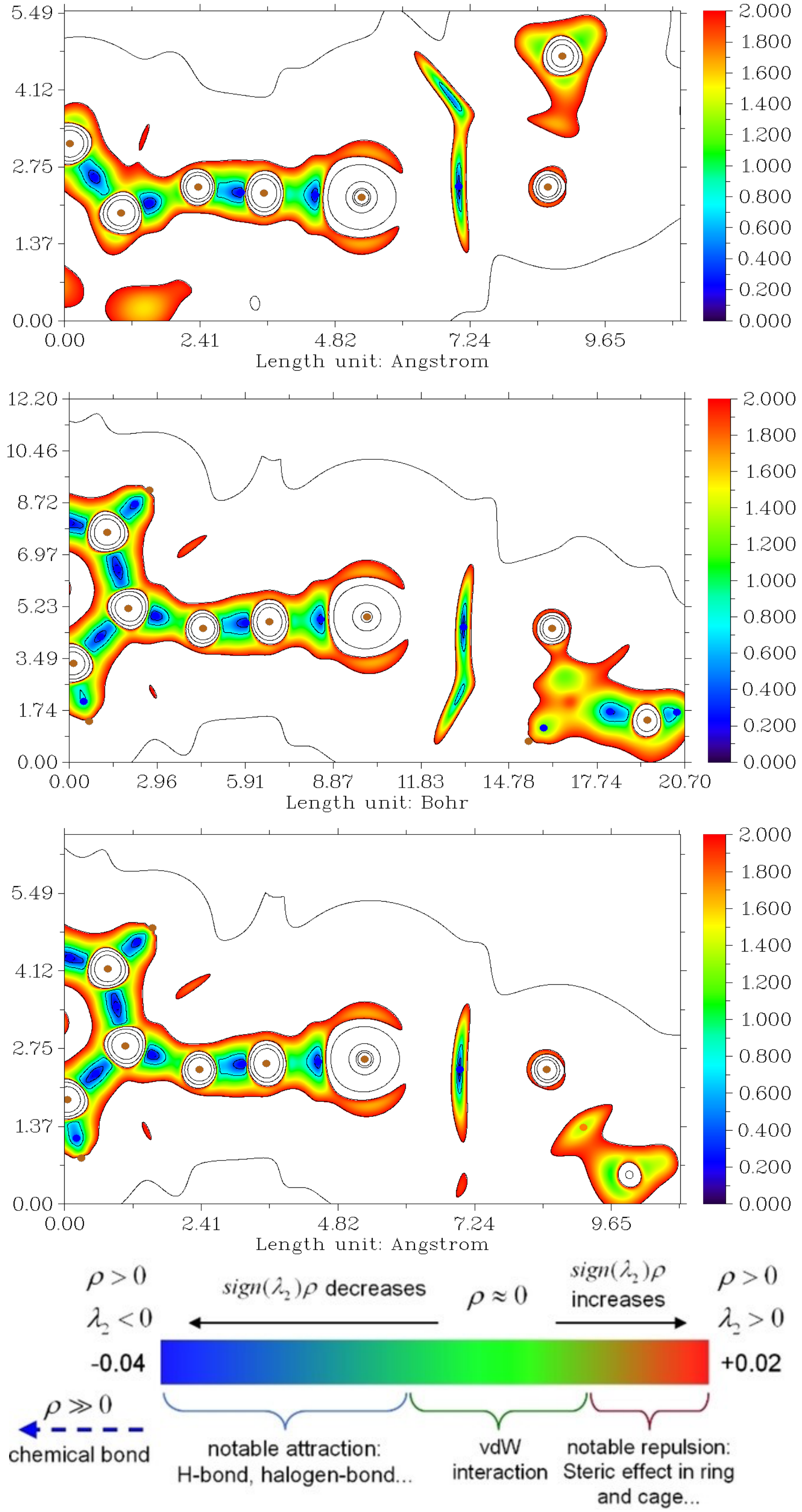

Figure S30. Interaction region indicator (IRI) maps for the interaction of isoselenocyanates with THF.

Table S11. Experimental vs. calculated ^{77}Se NMR chemical shifts (ppm; relative to IEF-PCM solvated $SeMe_2$); PheBoc: Boc-Phe-*Ψ*[CH_2NCSe],[22] ValBoc: Boc-Val-*Ψ*[CH_2NCSe],[22] Leuc: *Z*-Leu-*Ψ*[CH_2NCSe],[22] Fer: 1,1-diisoselenocyanatoferrocene.[23] Based on gas-phase optimized geometries at B3LYP/def2tzvp level; GIAO NMR prediction at B3LYP/x2c-TZVPPall-s level.

	Exp. ^{77}Se NMR (ppm)			Calc. ^{77}Se NMR (ppm)	
	$CDCl_3$	PhH/Tol	THF	Gas	IEF-PCM
SeCNFer		-365.3		-321	-466
SeCNLeuc	-358.1			-387	-530
SeCNPheBoc	-356.3			-387	-529
SeCNValBoc	-355.7			-386	-528
SeCN^{t}Bu	–345.5		–353.3	-392	-533
SeCNMesityl	–314.4		–325.3	-330	-478
SeCN$^{i\text{-Prop2}}$Ph	–311.2		–314.9	-326	-471
SeCNAr$^{\text{Mes2}}$	–294.3	–293.5	–301.3	-310	-463
SeCNPhF	–294.3		–304.6	-289	-437
SeCNPh	–294.6		–301.2	-292	-442
SeCNPhOMe	-302.3			-289	-439
SeCNAr$^{\text{Dipp2}}$	-288.5		-320.0	-298	-448
SeCNAr$^{\text{Tripp2}}$	–283.8	–286.1	–319.2	-302	-450
SeCN*p*-FAr$^{\text{DarF2}}$	–274.1	–276.4	–289.9	-275	-431

a)

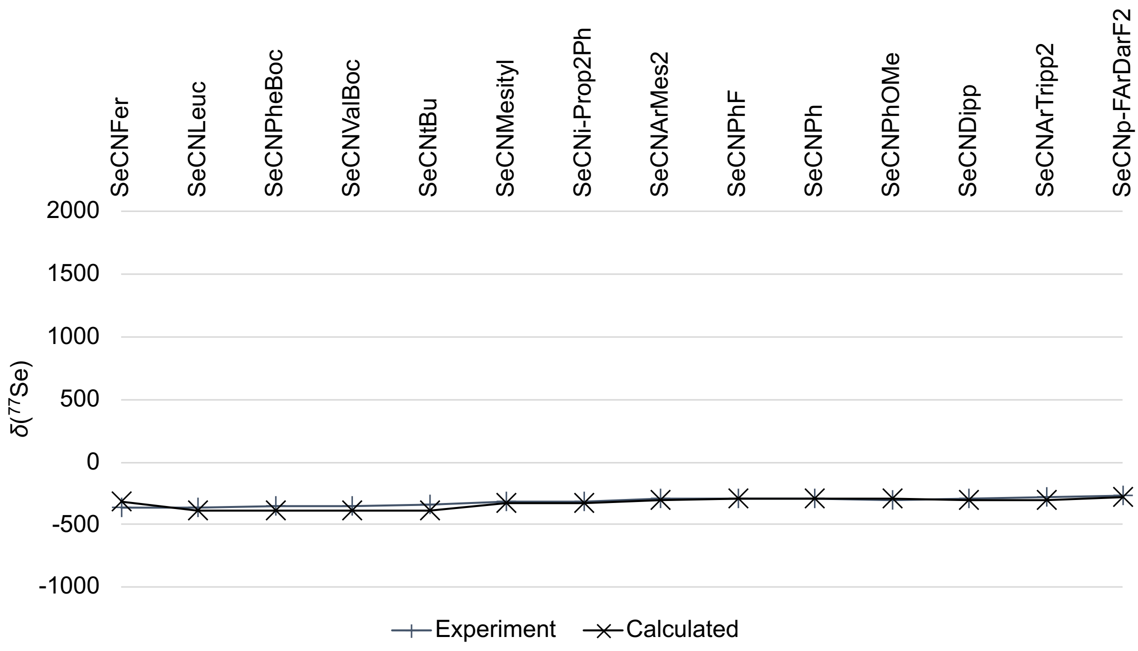

b)

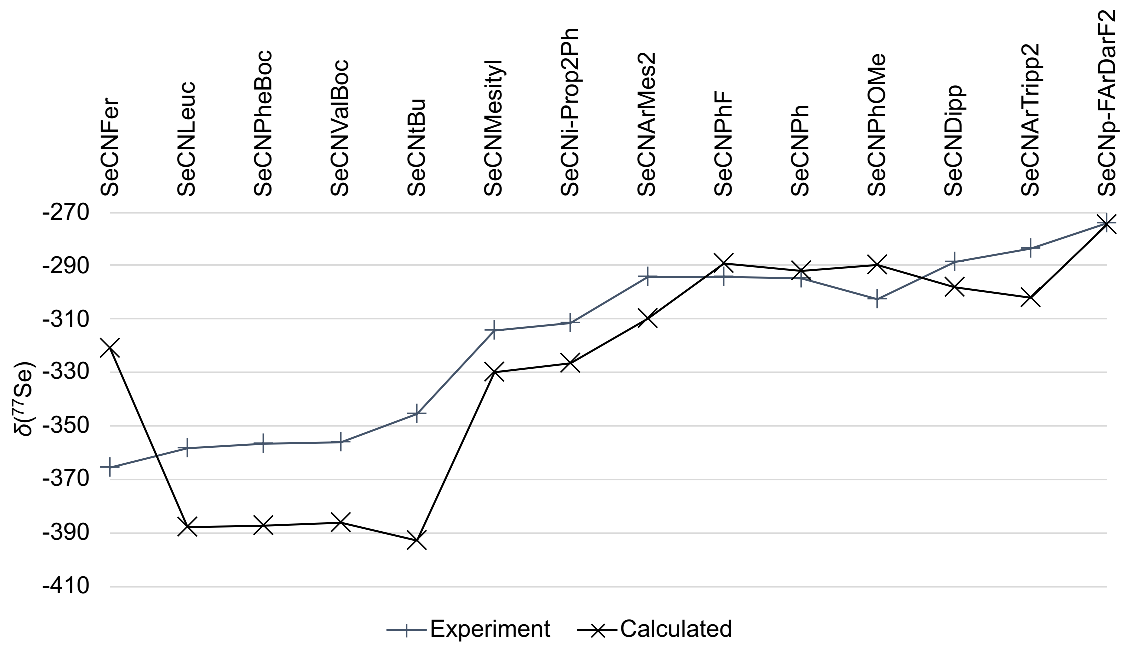

Figure S31. Graphical representations of the experimental ^{77}Se chemical shifts in $CHCl_3$ and the values of the gas phase simulations contained in Table S11. a) In the common spectral range of ^{77}Se NMR spectroscopy and b) as zoom into the region of interest indicating the deviations of the simulated data.

Table S12. Experimental vs. calculated ^{77}Se NMR chemical shifts (ppm; relative to CN^{t}Bu); PheBoc: Boc-Phe-*Ψ*[CH$_2$NCSe],[22] ValBoc: Boc-Val-*Ψ*[CH$_2$NCSe],[22] Leuc: *Z*-Leu-*Ψ*[CH$_2$NCSe],[22] Fer: 1,1-diisoselenocyanatoferrocene.[23] Based on gas-phase optimized geometries at B3LYP/def2tzvp level; GIAO NMR prediction at B3LYP/x2c-TZVPPall-s level. MAE: mean absolute error; R²: correlation coefficient.

	Exp. ^{77}Se NMR (ppm)		Calc. ^{77}Se NMR (ppm)		
	Unpolar3	$\delta_{solv.}$(THF)	Gas	IEF-PCM	$\Delta_{solv.}$
SeCNFer	-19.80		72	67	4.9
SeCNLeuc	-12.60		5	3	2.3
SeCNPheBoc	-10.80		5	4	1.4
SeCNValBoc	-10.20		6	5	1.3
SeCN^{t}Bu	0.00	7.8	0	0	0.0
SeCNMesityl	31.10	10.9	63	55	7.9
SeCN$^{i\text{-}Prop2}$Ph	34.30	3.7	66	62	3.9
SeCNAr^{Mes2}	51.20	7.0	83	70	12.7
SeCNPhF	51.20	10.3	104	96	7.4
SeCNPh	50.90	6.6	100	91	9.8
SeCNPhOMe	43.20		103	94	8.8
SeCNAr^{Dipp2}	57.00	31.5	94	85	9.5
SeCNAr^{Tripp2}	61.70	2.3	91	83	7.9
SeCN*p*-FAr^{DarF2}	71.40	15.8	118	102	15.5
MAE(ppm)/R²			36/0.72	30/0.70	3.1

Table S13. Comparison between different DFT work-flows; experimental vs. calculated ^{77}Se NMR chemical shifts (ppm; relative to CN^{t}Bu); PheBoc: Boc-Phe-*Ψ*[CH$_2$NCSe],[22] ValBoc: Boc-Val-*Ψ*[CH$_2$NCSe],[22] Leuc: *Z*-Leu-*Ψ*[CH$_2$NCSe],[22] Fer: 1,1-diisoselenocyanatoferrocene.[23] Based on gas-phase optimized geometries at B3LYP/def2tzvp level; GIAO NMR prediction at B3LYP/x2c-TZVPPall-s level. MAE: mean absolute error; R²: correlation coefficient.

Geometry optimization	NMR calc.	SeCN^{t}Bu	SeCNPh
**Experiment, unpolar**		**0**	**50.9**
**Experiment, THF**		**0**	**57.5**
Gas-phase	Gas-phase	0	100
Gas-phase	IEF-PCM: CH_2Cl_2	0	91
Gas-phase	IEF-PCM: THF	0	91
IEF-PCM: CH_2Cl_2	Gas-phase	0	100
IEF-PCM: CH_2Cl_2	IEF-PCM: CH_2Cl_2	0	84
IEF-PCM: THF	Gas-phase	0	100
IEF-PCM: THF	IEF-PCM: CH_2Cl_2	0	84

Table S14. Calculated free energies for isoselenocyanide formation from isocyanides and elemental selenium in the gas-phase. PheBoc: Boc-Phe-*Ψ*[CH$_2$NCSe],[22] ValBoc: Boc-Val-*Ψ*[CH$_2$NCSe],[22] Leuc: *Z*-Leu-*Ψ*[CH$_2$NCSe],[22] Fer: 1,1-diisoselenocyanatoferrocene.[23]

	Δ*G* [Hartree]		|ΔΔ*G*| [kJ/mol]
	SeCNR	CNR	SeCNR *vs.* CNR + 1/8 Se_8
SeCNFer	-6638.603	-1835.35	37
SeCNLeuc	-3245.257	-843.63	31
SeCNPheBoc	-3245.262		32
SeCNValBoc	-3092.804	-691.18	31
SeCN^{t}Bu	-2652.293	-250.67	34
SeCNMesityl	-2844.064	-442.44	38
SeCN$^{i\text{-Prop2}}$Ph	-2961.955	-560.33	37
SeCNAr^{Mes2}	-3424.096		40
SeCNPhF	-2825.431	-423.80	38
SeCNPh	-2726.142	-324.51	38
SeCNPhOMe	-2840.683	-439.05	38
SeCNAr^{Dipp2}	-3659.878	-1494.07	39
SeCNAr^{Tripp2}	-3895.695	-1285.25	41
SeCN*p*-FAr^{DarF2}	-4636.333	-2234.70	51
SeCO	-2514.994	-113.377	9

Table S15. Calculated free energies for diverse references and mechanistically interesting hypothetical molecules.

	Δ*G* [Hartree]	|ΔΔ*G*| [kJ/mol]
Se_8	-19212.914	
1/8 Se_8	-2401.614	
NEt_3	-292.357	
1/8 Se_8 + NEt_3	-2693.971	129
$^-Se-^+NEt_3$	-2693.922	
CN^{t}Bu + $^-Se-^+NEt_3$	-2944.580	163
SeCN^{t}Bu + NEt_3	-2944.650	
SeCN^{t}Bu	-2652.293	245
cyclo-C(Se)N^{t}Bu	-2652.199	

Table S16. Calculated free energies for the nucleophilic attack of HN(*i*-Prop)$_2$ at SeCN*p*-FAr^{DarF2} forming a selenourea.

	Gas phase	IEF-PCM THF
Δ*G*(Selenourea) [Hartree]	-4928.672	-4928.685
Δ*G*(SeCN*p*-FAr^{DarF2}+ HN(*i*-Prop)$_2$; σ-hole) [Hartree]	-4928.683	-4928.693
Δ*G*(SeCN*p*-FAr^{DarF2}+ HN(*i*-Prop)$_2$; side-on N-H···NC) [Hartree]	-4928.684	-4928.693
ΔΔ*G* [kJ/mol]	33	23
